# Healthy and unhealthy plant‐based diets in relation to the incidence of colorectal cancer overall and by molecular subtypes

**DOI:** 10.1002/ctm2.893

**Published:** 2022-08-23

**Authors:** Fenglei Wang, Tomotaka Ugai, Koichiro Haruki, Yi Wan, Naohiko Akimoto, Kota Arima, Rong Zhong, Tyler S. Twombly, Kana Wu, Kanhua Yin, Andrew T. Chan, Marios Giannakis, Jonathan A. Nowak, Jeffrey A. Meyerhardt, Liming Liang, Mingyang Song, Stephanie A. Smith‐Warner, Xuehong Zhang, Edward L. Giovannucci, Walter C. Willett, Shuji Ogino

**Affiliations:** ^1^ Department of Nutrition Harvard T.H. Chan School of Public Health Boston Massachusetts; ^2^ Program in MPE Molecular Pathological Epidemiology Department of Pathology Brigham and Women's Hospital and Harvard Medical School Boston Massachusetts; ^3^ Department of Epidemiology Harvard T.H. Chan School of Public Health Boston Massachusetts; ^4^ Department of Medical Oncology Dana‐Farber Cancer Institute and Harvard Medical School Boston Massachusetts; ^5^ Department of Gastroenterological Surgery Graduate School of Medical Sciences Kumamoto University Kumamoto Japan; ^6^ Department of Epidemiology and Biostatistics and Ministry of Education Key Lab of Environment and Health School of Public Health Tongji Medical College Huazhong University of Science and Technology Wuhan Hubei China; ^7^ Department of Surgery Massachusetts General Hospital Boston Massachusetts; ^8^ Channing Division of Network Medicine Brigham and Women's Hospital and Harvard Medical School Boston Massachusetts; ^9^ Clinical and Translational Epidemiology Unit Massachusetts General Hospital and Harvard Medical School Boston Massachusetts; ^10^ Division of Gastroenterology Massachusetts General Hospital Boston Massachusetts; ^11^ Broad Institute of MIT and Harvard University Cambridge Massachusetts; ^12^ Department of Medicine Brigham and Women's Hospital and Harvard Medical School Boston Massachusetts; ^13^ Department of Biostatistics Harvard T.H. Chan School of Public Health Boston Massachusetts; ^14^ Cancer Immunology and Cancer Epidemiology Programs Dana‐Farber Harvard Cancer Center Boston Massachusetts

**Keywords:** colorectal carcinoma, inverse probability weighting, molecular pathological epidemiology, sustainability

## Abstract

**Background:**

Plant‐based foods have been recommended for health. However, not all plant foods are healthy, and little is known about the association between plant‐based diets and specific molecular subtypes of colorectal cancer (CRC). We examined the associations of healthy and unhealthy plant‐based diets with the incidence of CRC and its molecular subtypes.

**Methods:**

While 123 773 participants of the Nurses’ Health Study and the Health Professionals Follow‐up Study had been followed up (3 143 158 person‐years), 3077 of them had developed CRC. Healthy and unhealthy plant‐based diet indices (hPDI and uPDI, respectively) were calculated using repeated food frequency questionnaire data. We determined the tumoural status of microsatellite instability (MSI), CpG island methylator phenotype (CIMP), and *BRAF* and *KRAS* mutations.

**Results:**

Higher hPDI was associated with lower CRC incidence (multivariable hazard ratio [HR] comparing extreme quartiles, 0.86, 95% confidence interval [CI]: 0.77, 0.96; *P*‐trend = .04), whereas higher uPDI was associated with higher CRC incidence (multivariable HR comparing extreme quartiles, 1.16, 95% CI: 1.04, 1.29; *P*‐trend = .005). The association of hPDI significantly differed by *KRAS* status (*P*‐heterogeneity = .003) but not by other tumour markers. The hPDI was associated with lower incidence of *KRAS*‐wildtype CRC (multivariable HR comparing extreme quartiles, 0.74, 95% CI: 0.57, 0.96; *P*‐trend = .004) but not *KRAS*‐mutant CRC (*P*‐trend = .22).

**Conclusions:**

While unhealthy plant‐based diet enriched with refined grains and sugar is associated with higher CRC incidence, healthy plant‐based diet rich in whole grains, fruits and vegetables is associated with lower incidence of CRC, especially *KRAS*‐wildtype CRC.

## Graphical abstract

• An unhealthy plant‐based diet rich in refined grains and sugar is associated with higher CRC incidence.

• A healthy plant‐based diet rich in whole grains, fruits and vegetables is associated with lower incidence of CRC, especially *KRAS*‐wildtype CRC.

• Replacing refined grains with healthy plant foods such as whole grains, fruits and vegetables is associated with lower CRC incidence.

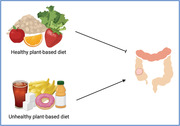

## 1 INTRODUCTION

Colorectal adenocarcinomas remain to be the second most common cause of cancer death in the world.^1^ It is estimated that eliminating the effect of poor‐quality diet in the United States may reduce colorectal cancer (CRC) incidence by approximately 38%.^2^ Western‐style diet, especially high in red and processed meats, is associated with increased CRC incidence.^3^ Besides the carcinogenic compounds (such as N‐nitroso compounds, heterocyclic amines and polycyclic aromatic hydrocarbons), other nutrients enriched in meats, including haeme iron, sulphur and choline, can also contribute to the development of CRC.^4^

Several plant‐based foods and nutrients, including whole grains, fruits, vegetables and fibre, have been associated with a lower CRC risk.^5^, ^6^, ^7^ The latest scientific report from the 2020 US Dietary Guidelines Advisory Committee identified whole grains, fruits and vegetables as three fundamental constituents of a healthy dietary pattern.^8^ The production of these foods is in general environmentally more sustainable (i.e. environmentally more friendly) than animal‐based foods. However, not every plant‐based food is good for consumer's health. Less nutrient‐dense plant‐based foods, including refined grains and sugar‐sweetened beverages (such as carbonated beverages with sugar), are associated with higher risks of cardiometabolic diseases^9^, ^10^ and CRC.^11^, ^12^ Thus, it is essential to differentiate between healthy and unhealthy plant foods when advocating plant‐based diets for CRC prevention.

Additionally, the diet‐CRC association may differ according to various molecular subtypes.^13^, ^14^ Certain tumour molecular characteristics, such as CpG island methylator phenotype (CIMP), microsatellite instability (MSI) and somatic mutations in *BRAF* and *KRAS*, have been widely investigated with regard to the heterogeneity of diet and CRC association.^14^, ^15^, ^16^, ^17^, ^18^, ^19^, ^20^, ^21^ However, most of these studies primarily focused on an individual food item or nutrient. Data are limited on the heterogeneity in the association between dietary patterns and the incidence of CRC subclassified by molecular subtypes. Therefore, we conducted a prospective study to test hypotheses that healthy and unhealthy plant‐based dietary patterns might be associated with CRC incidence and that the association might differ by individual tumour molecular subtypes or in combination.

## 2 METHODS

### 2.1 Study population

**FIGURE 1:**
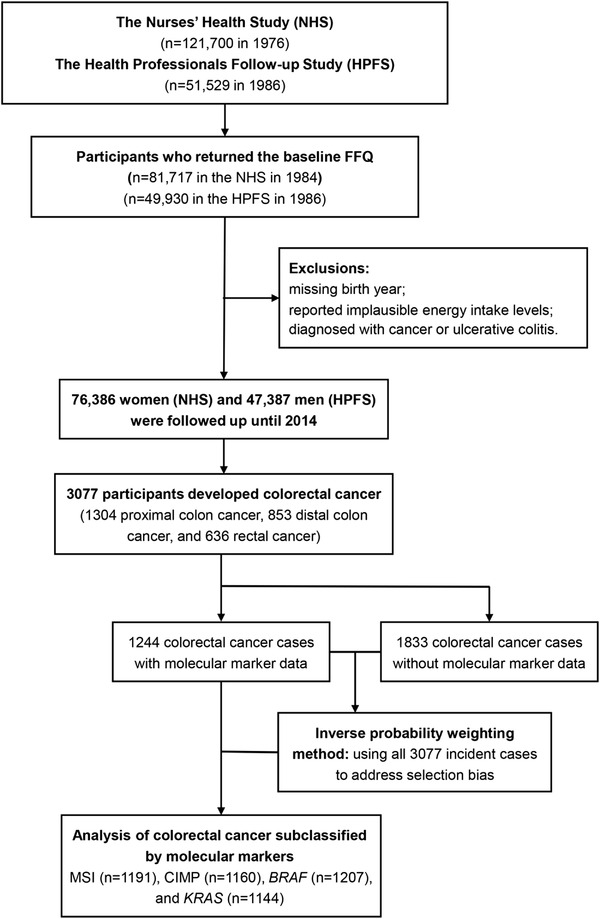
Flow chart of study population. CIMP, CpG island methylator phenotype; FFQ, food frequency questionnaire; MSI, microsatellite instability

The present study used data from two prospective cohorts, namely, the Nurses’ Health Study (NHS) and the Health Professionals Follow‐up Study (HPFS). The NHS enrolled 121 700 nurses who were 30‐year‐old to 55‐year‐old women in 1976.^22^ The HPFS recruited 51 529 health professionals who were 40‐year‐old to 75‐year‐old men in 1986.^22^ Every 2 years, the studies have sent detailed questionnaires to cohort participants to obtain information on lifestyle and health‐related conditions. We excluded participants who did not send answers to the baseline (1984 for the NHS and 1986 for the HPFS) food frequency questionnaire (FFQ), reported nearly impossible daily energy intake (<500 or >3500 kcal/day for women and < 800 or >4200 kcal/day for men), did not report their dates of birth, or reported past personal history of malignancy (except non‐melanoma cancer of skin) or ulcerative colitis before their enrolment. After these exclusions, data from 123 773 participants (76 386 women and 47 387 men) were utilised in the current analysis (Figure 1).

### 2.2 Examination of diets and the plant‐based diet indices

Dietary data were extracted from semi‐quantitative FFQs in 1984, 1986, 1990, 1994, 1998, 2002, 2006 and 2010 in the NHS, and 1986, 1990, 1994, 1998, 2002, 2006, 2010 and 2014 in the HPFS. The reproducibility and validity of the FFQs have been reported elsewhere.^23^, ^24^ Plant‐based diet indices were developed as described previously.^25^, ^26^ In brief, we first categorised all foods to 18 groups within three broad categories: healthy plant foods, including whole grains, fruits, vegetables, legumes, nuts, tea/coffee and vegetable oils; unhealthy plant foods, including refined grains, potatoes, sweets/desserts, fruit juice and sugar‐sweetened beverages and animal foods, including animal fats, meat, eggs, dairy, fish/seafood and miscellaneous animal foods. These food groups were categorised based on nutrient and culinary similarities. Healthy and unhealthy plant foods were distinguished using existing knowledge of associations of the foods with type 2 diabetes, cardiovascular disease, certain cancers and intermediate conditions (obesity, hypertension, hyperlipidemia and systemic inflammation).^26^ The 18 food groups were then divided into quintiles of consumption, and each quintile was assigned a score of 1 to 5. For the healthy plant‐based diet index (hPDI), positive scores (a score of 1 was assigned to the lowest quintile and 5 to the highest) were assigned to healthy plant food groups and reverse scores (a score of 5 was assigned to the lowest quintile and 1 to the highest) to unhealthy plant food groups; for the unhealthy plant‐based diet index (uPDI), positive scores were assigned to unhealthy plant food groups and reverse scores to healthy plant food groups. We also derived an overall plant‐based diet index (PDI), where both healthy and unhealthy plant foods were given positive scores. Reverse scores were assigned to animal food groups for all three indices. Finally, we summed up 18 food group scores to obtain the indices, each ranging from 18 to 90. These indices have been widely used in other cohorts.^27^, ^28^, ^29^, ^30^

### 2.3 Covariate assessment

We collected information on body weight, physical exercise activity, regular use of aspirin or other NSAID, smoking habits, family history of CRC, history of previous lower gastrointestinal endoscopic examination (and status of menopause and postmenopausal use of hormone therapy in women) through the baseline and biennial follow‐up questionnaires.^22^

### 2.4 Assessment of colorectal cancer cases

When participants had diagnosis of CRC, it was reported in biennial questionnaires. Unreported CRC cases, a vast majority of which were lethal CRCs, were identified through use of the National Death Index and questionnaire returned by next of kin. Study participants with CRC diagnosis (or their next of kin if participants with CRC were deceased) were asked for permission to examine medical records of the CRC participants. Study physicians, who were blinded to information on exposures, carefully examined all medical records to confirm the diagnosis of colorectal adenocarcinoma and obtain data on detailed colorectal tumour location and tumour‐node‐metastasis (TNM) stage. Both colon and rectal cancers were regarded as outcomes in the current study, in consideration of the colorectal continuum model.^31^

### 2.5 Tumour molecular analyses

We attempted to collect formalin‐fixed paraffin‐embedded (FFPE) tumour and normal tissue from all incident CRC cases in which we obtained consent for tissue analyses. The study pathologist (S.O.) conducted histopathological examinations and marked tumour‐rich areas in all cases with available tissue. Genomic DNA was extracted from tumour and normal tissues. The quantity and quality of DNA specimens extracted from FFPE tissue have been shown to be stable for up to 12 years.^32^ We analysed four well‐studied colorectal tumour molecular characteristics: microsatellite instability (MSI), CpG island methylator phenotype (CIMP), and *BRAF* and *KRAS* mutations. Polymerase chain reaction (PCR) followed by pyrosequencing were done on *BRAF* codon 600 and *KRAS* codons 12, 13, 61 and 146 (Supplementary Table 1).^33^, ^34^ MSI status was analysed by PCR assays of 10 microsatellite markers (BAT25, BAT26, BAT40, D17S250, D18S55, D18S56, D18S67, D18S487, D2S123 and D5S346). MSI‐high tumours were defined as tumours with instability in ≥30% of the markers.^31^ We quantified DNA methylation levels using bisulphite modification followed by real‐time quantitative PCR (MethyLight)^35^ for 8 CIMP‐specific promoters (*CACNA1G*, *CDKN2A* [p16], *CRABP1*, *IGF2*, *MLH1*, *NEUROG1*, *RUNX3* and *SOCS1*)^36^ and classified tumours as CIMP‐high if ≥6 promoters were methylated and CIMP‐low/negative if 0 to 5 promoters were methylated.^37^

We also used a colorectal carcinoma classification system using a combination of four biomarkers: Type 1 (MSI‐high, CIMP‐high, *BRAF* mutant, *KRAS *wild‐type), Type 2 (non‐MSI‐high, CIMP‐high, *BRAF* mutant, *KRAS *wild‐type), Type 3 (non‐MSI‐high, CIMP‐low/negative, *BRAF* wild‐type, *KRAS*‐mutant), Type 4 (non‐MSI‐high, CIMP‐low/negative, *BRAF *wild‐type, *KRAS *wild‐type) and Type 5 (MSI‐high, CIMP‐low/negative, *BRAF *wild‐type, *KRAS *wild‐type).^38^ These five combinatorial subtype have been related to the three different etiological pathways: (a) serrated pathway [Type 1 and Type 2], (b) alternate pathway [Type 3] and (c) conventional adenoma pathway [Type 4 and Type 5].^14^, ^38^

### 2.6 Statistical analysis

We conducted restricted cubic spline analyses to test the possible non‐linear relationships of hPDI and uPDI with overall CRC risk, and no spline variables were added into the model, suggesting no substantial departure from linearity. Therefore, we set our primary hypothesis testing to assess the statistical linear trend for the association of hPDI (or uPDI) with overall CRC, as well as the heterogeneity of the linear trend for the association of each index with CRC incidence subclassified by either of the four molecular markers. All other tests were considered as secondary analyses, to reduce the number of primary hypothesis tests. Furthermore, we used the stringent two‐sided α level of .005 was employed as a stringent significance level which has been set by expert statisticians.^39^

We used time (months) in following each participant from the return date of the baseline FFQ until diagnosis of CRC, death, or end of follow‐up (June 30, 2014, for NHS and January 31, 2014, for HPFS), whichever first had come. As no substantial or significant heterogeneity (using Q‐statistic) between the two cohorts was observed for the association of hPDI, uPDI, or overall PDI with CRC incidence, we pooled individual‐level data from both cohorts for further analyses. We employed multivariable‐adjusted time‐varying Cox proportional hazards regression models (which were statistically stratified by age, cohort and calendar year) to compute the hazard ratio (HR) for CRC incidence.

To assess the long‐term habitual dietary intake patterns, we used the cumulative average of each plant‐based diet index from the baseline FFQ up to the start of each follow‐up interval before CRC diagnosis, death, or end of follow‐up. The cumulative average of the index was categorised into study‐specific quartiles. We also used the cumulative average for body mass index, physical activity and dietary covariates (alcohol intake and total energy intake). Multivariable models were adjusted for body mass index (continuous with a ceiling at 35 kg/m^2^),^40^ physical activity (continuous with a ceiling at 50 metabolic equivalent task score‐hours/week),^40^ regular use of aspirin or other non‐steroidal anti‐inflammatory drugs (≥2 tablets/week: yes or no), smoking status (never, past or current), family history of CRC (yes or no), history of previous lower gastrointestinal endoscopy (yes or no), alcohol intake (continuous with a ceiling at 30 g/day) and total energy intake (continuous). Analyses of only women (i.e. NHS without the HPFS) were adjusted for postmenopausal hormone use (premenopausal, postmenopausal never, past, or current use) in addition to the aforementioned variables. For missing data in covariates (missing proportion 0.4% for body mass index and for 2.4% physical activity), we carried forward the value collected in the closest questionnaire cycle with available data. The proportional hazards assumption was tested by adding interaction terms between follow‐up time and plant‐based diet indices, demonstrating no evidence for statistically significant deviation from the assumption.

When testing for linear trend, to maximise the use of the data and minimise the influence of extreme values, we placed lower and upper ceilings at the 5th and 95th percentiles, respectively, of each index (Supplementary Figure 1) (with values below the 5th or above the 95th percentile being replaced by the 5th or 95th percentile value, respectively) and put it into the regression model as a continuous term. One recently published paper indicated that the metabolic health effects of plant‐based diets were driven by the total protein amount rather than the plant versus animal source in the diet.^41^ To test if this was applicable to the health benefits on CRC prevention, we examined the associations of total protein, total fat and protein/fat from plant or animal source with CRC incidence. In addition, we evaluated the associations of the individual plant food groups with CRC incidence while adjusting for six animal food groups and covariates for indices analysis. We also estimated the associations of substituting equal servings of whole grains, fruits or vegetables for refined grains, as we found refined grains were the major unhealthy plant food group that was correlated with higher CRC incidence. The substitution analyses were conducted by including both food groups as continuous variables in the same multivariable model, which also contained total energy intake and other covariates. The difference of the parameter estimates of the two food groups and the corresponding variances and covariance were then used to estimate substitution associations.^42^

We adopted Cox proportional hazards regression models with competing risks data duplication method that could assess whether the association of hPDI (or uPDI) with CRC incidence differed according to tumour location or molecular subtype. Heterogeneity was tested using a likelihood ratio test that compared a model allowing for separate associations with CRC subtypes to another model presuming a common association with the CRC subtypes.^43^ Given that not all CRC cases provided tissue materials for tumour molecular biomarker assessments, inverse probability weighting (IPW) was employed to control for selection bias due to the variable availability of tissue biomarker data.^44^ Cox regression analyses without using IPW were conducted as a sensitivity analysis.

We conducted other sensitivity analyses by stopping any further updates to diet after diagnosis of other morbidity outcomes that might change a person's dietary habits (diabetes, cardiovascular diseases and cancers other than CRC), to test the robustness of our findings. All analyses were conducted using SAS software version 9.4 (SAS Institute, Cary, North Carolina, USA).

## 3 RESULTS

While 123 773 study subjects in the two cohorts had been followed up (3 143 158 person‐years), a total of 3077 participants had been found to have diagnosis of colorectal cancer (CRC). The healthy plant‐based diet index (hPDI) was associated positively with physical activity and negatively with smoking (Table 1), whereas the unhealthy plant‐based diet index (uPDI) was associated positively with smoking and negatively with physical activity (Supplementary Table 2). The hPDI, uPDI and overall plant‐based diet index (PDI) were generally stable during the follow‐up period (Supplementary Figure 2).

**TABLE 1:** Age‐standardised characteristics of participants in the Nurses’ Health Study and the Health Professionals Follow‐up Study, according to quartiles of the healthy plant‐based diet index

	Nurses’ Health Study				Health professionals follow‐up study			
	Quartile 1	Quartile 2	Quartile 3	Quartile 4	Quartile 1	Quartile 2	Quartile 3	Quartile 4
Person‐years	515 401	507 822	517 637	510 928	274 459	266 807	271 500	278 604
Age at baseline, years (mean)	48	49	50	52	51	52	53	54
Body mass index, kg/m^2^ (mean)	26.3	26.0	25.7	25.1	25.7	25.7	25.5	25.1
Physical activity, METS‐hour/week (mean)	13.4	15.1	16.8	20.1	28.2	29.4	31.1	34.7
Current smoker (%)	15	13	12	11	8	7	6	4
Non‐drinker of alcohol (%)	24	23	23	24	18	17	17	18
History of previous endoscopy (%)	23	23	23	23	32	34	35	36
Family history of colorectal cancer (%)	19	19	19	19	15	15	15	15
Regular use of aspirin or other non‐steroidal anti‐inflammatory drugs (%)	34	34	33	32	35	37	37	36
Premenopausal (%)	12	12	12	11	/	/	/	/
Current postmenopausal hormone use (%)	23	25	26	28	/	/	/	/
Dietary intake (mean)								
Alcohol, among drinkers, g/day	7.8	7.9	7.8	7.7	13.2	13.5	13.6	13.1
Total energy, kcal/day	1991	1788	1654	1523	2278	2019	1864	1743
Total dietary fibre, g/day	14.6	16.6	18.3	21.6	17.7	20.3	22.6	27.7
Total folate, µg/day	401	438	468	521	474	523	562	633
Healthy plant foods								
Whole grains, serving/day	1.0	1.2	1.4	1.6	1.2	1.5	1.7	2.1
Fruits, serving/day	1.2	1.5	1.6	2.0	1.3	1.5	1.7	2.2
Vegetables, serving/day	2.6	3.0	3.2	3.8	2.6	2.9	3.2	3.8
Legumes, serving/week	2.5	2.7	2.8	3.2	2.7	2.9	3.1	3.8
Nuts, serving/week	1.4	1.6	1.7	1.9	2.3	2.5	2.6	3.0
Vegetable oils, serving/week	1.6	1.9	2.2	2.9	1.6	1.9	2.2	2.8
Tea/coffee, serving/day	2.6	2.9	3.0	3.1	2.1	2.3	2.4	2.4
Unhealthy plant foods								
Refined grains, serving/day	2.1	1.7	1.4	1.1	2.0	1.6	1.4	1.2
Sweets/desserts, serving/day	1.7	1.3	1.1	0.8	2.0	1.5	1.2	0.9
Potatoes, serving/week	4.5	3.6	3.0	2.3	5.0	4.0	3.4	2.7
Fruit juice, serving/week	6.2	5.4	4.8	3.9	6.4	5.7	5.2	4.6
Sugar‐sweetened beverages, serving/week	3.5	2.0	1.2	0.6	4.4	2.5	1.7	0.8
Animal foods								
Animal fats, serving/week	4.2	2.4	1.6	1.0	3.4	1.8	1.2	0.7
Dairy products, serving/day	2.3	2.1	2.0	1.8	2.3	2.0	1.8	1.5
Eggs, serving/week	2.5	2.1	1.8	1.5	2.8	2.2	1.8	1.3
Fish/seafood, serving/week	2.1	2.2	2.2	2.3	2.5	2.6	2.7	2.8
Meat, serving/day	1.8	1.6	1.5	1.2	2.2	1.8	1.6	1.2
Miscellaneous animal foods, serving/week	3.5	3.0	2.6	2.0	3.6	2.9	2.4	1.8
Healthy plant‐based diet index (mean)	46.9	52.8	57.0	63.0	46.4	52.6	57.0	63.5
Unhealthy plant‐based diet index (mean)	57.8	55.6	54.0	51.6	57.1	55.4	54.2	52.1

*Note*: All variables are standardised to the age distribution of the study population, except for age at baseline.

Abbreviation: METS, metabolic equivalent task score.

A higher hPDI was associated with lower incidence of CRC (multivariable *P*‐trend = .04), while a higher uPDI was associated with increased CRC incidence (multivariable *P*‐trend = .005) (Table 2). Multivariable HR for participants in the highest hPDI quartile compared to those in the lowest quartile was 0.86 (95% confidence interval [CI]: 0.77, 0.96). In contrast, multivariable HR for participants in the highest uPDI quartile compared to those in the lowest quartile was 1.16 (95% CI: 1.04, 1.29).

**TABLE 2:** Hazard ratios with 95% confidence intervals of incident colorectal cancer according to the healthy or unhealthy plant‐based diet index in the Nurses’ Health Study (NHS) and the Health Professionals Follow‐up Study (HPFS)^a^

	Quartiles of healthy or unhealthy plant‐based diet index				
	Quartile 1	Quartile 2	Quartile 3	Quartile 4	*P*‐trend^b^
**Healthy plant‐based diet index**					
**NHS**					
No. of cases	400	386	463	439	
Age‐adjusted	1 (reference)	0.88 (0.77, 1.02)	0.97 (0.84, 1.11)	0.86 (0.75, 0.99)	.08
Multivariable‐adjusted	1 (reference)	0.90 (0.78, 1.04)	1.00 (0.87, 1.15)	0.92 (0.79, 1.06)	.46
**HPFS**					
No. of cases	339	328	358	364	
Age‐adjusted	1 (reference)	0.86 (0.73, 1.00)	0.85 (0.73, 0.99)	0.79 (0.68, 0.92)	.009
Multivariable‐adjusted	1 (reference)	0.86 (0.73, 1.01)	0.86 (0.73, 1.01)	0.82 (0.70, 0.98)	.07
**Pooled**					
No. of cases	739	714	821	803	
Age‐adjusted	1 (reference)	0.87 (0.79, 0.97)	0.91 (0.83, 1.01)	0.83 (0.75, 0.92)	.002
Multivariable‐adjusted	1 (reference)	0.88 (0.79, 0.97)	0.93 (0.83, 1.03)	0.86 (0.77, 0.96)	.04
**Unhealthy plant‐based diet index**					
**NHS**					
No. of cases	401	426	416	445	
Age‐adjusted	1 (reference)	1.08 (0.94, 1.24)	1.05 (0.91, 1.20)	1.16 (1.01, 1.33)	.05
Multivariable‐adjusted	1 (reference)	1.08 (0.94, 1.24)	1.03 (0.90, 1.19)	1.14 (0.98, 1.32)	.14
**HPFS**					
No. of cases	357	345	356	331	
Age‐adjusted	1 (reference)	1.00 (0.86, 1.16)	1.06 (0.91, 1.23)	1.05 (0.90, 1.23)	.29
Multivariable‐adjusted	1 (reference)	1.04 (0.89, 1.21)	1.12 (0.95, 1.30)	1.14 (0.96, 1.34)	.05
**Pooled**					
No. of cases	758	771	772	776	
Age‐adjusted	1 (reference)	1.04 (0.94, 1.15)	1.05 (0.95, 1.17)	1.11 (1.00, 1.23)	.03
Multivariable‐adjusted	1 (reference)	1.07 (0.96, 1.18)	1.08 (0.97, 1.20)	1.16 (1.04, 1.29)	.005

^a^ All analyses were stratified by age (in month), calendar year and sex. Multivariable‐adjusted hazard ratios were adjusted for body mass index (continuous with a ceiling at 35 kg/m^2^), physical activity (continuous with a ceiling at 50 metabolic equivalent task score‐hours/week), smoking status (never, past, or current), regular use of aspirin or other non‐steroidal anti‐inflammatory drugs (≥2 tablets per week: yes or no), family history of colorectal cancer (yes or no), history of previous lower gastrointestinal endoscopy (yes or no), alcohol intake (continuous with a ceiling at 30 g/day) and total energy intake (continuous). In NHS‐only analyses, we also adjusted for postmenopausal hormone use (premenopausal, postmenopausal never, past, or current use).

^b^ The healthy (or unhealthy) plant‐based diet index was used as a continuous variable in the regression model except for individuals below 5th percentile and those above 95th percentile for whom the 5th and 95th percentile values, respectively, were used to eliminate outlier effects.

Abbreviations: HPFS, Health Professionals Follow‐up Study; NHS, Nurses’ Health Study.

There was little evidence for heterogeneity between the two cohorts (*P*‐heterogeneity = .43 for hPDI; and *P*‐heterogeneity = .59 for uPDI). We did not observe evidence of heterogeneity in the association of hPDI or uPDI with CRC incidence by tumour locations in each cohort separately or in the pooled combined cohorts (Supplementary Table 3). The PDI was not associated with CRC incidence (multivariable *P*‐trend = .59) (Supplementary Table 4). Participants had similar average fish/seafood intake across quartiles of hPDI, whereas participants with higher uPDI tended to have lower fish/seafood intake (Table 1). Thus, we further adjusted for fish/seafood intake and found that the results remained similar (Supplementary Table 5).

**FIGURE 2:**
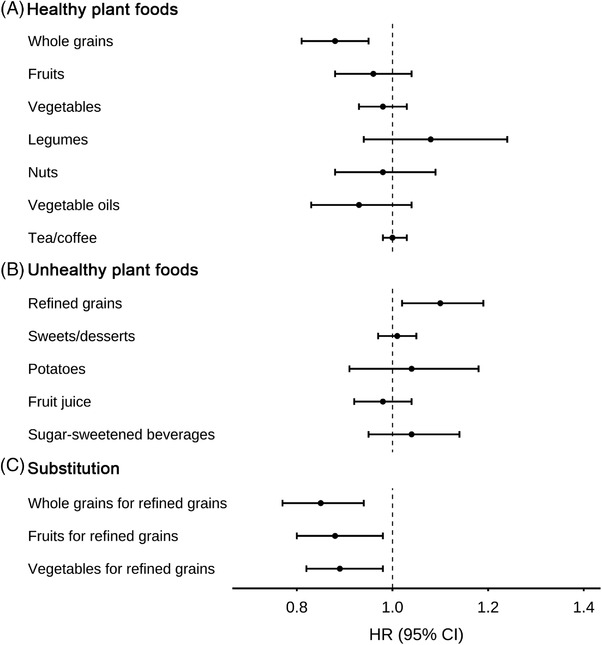
Association of individual plant food with colorectal cancer risk in the pooled cohort of Nurses’ Health Study and the Health Professionals Follow‐up Study. (A) Associations for healthy plant foods and (B) unhealthy plant foods. (C) Associations by equally substituting whole grains, fruits, or vegetables for two servings of refined grains. The associations in (A) and (B) were two servings/day for whole grains, fruits, vegetables and refined grains, and one serving/day for all other plant foods. All models were stratified by age (in month), calendar year and sex and adjusted for body mass index (continuous with a ceiling at 35 kg/m^2^), physical activity (continuous with a ceiling at 50 metabolic equivalent task score‐hours/week), smoking status (never, past, or current), regular use of aspirin or other non‐steroidal anti‐inflammatory drugs (≥2 tablets per week: yes or no), family history of colorectal cancer (yes or no), history of previous lower gastrointestinal endoscopy (yes or no), alcohol intake (continuous with a ceiling at 30 g/day), total energy intake (continuous) and intake of six animal food groups (continuous)

We did not observe any significant associations of total protein or total fat intake, or their intakes from plant or animal source and CRC incidence (Supplementary Table 6). Analysis of individual plant food groups showed that whole grains intake was associated with lower incidence of CRC (multivariable HR for a unit increase of two servings/day, 0.88, 95% CI: 0.81, 0.95; *p* = .001) (Figure 2A). In contrast, refined grains intake was associated with higher CRC incidence (multivariable HR for a unit increase of two servings/day, 1.10, 95% CI: 1.02, 1.19; *p* = .01) (Figure 2B). In substitution analyses in which two servings/day of refined grains were replaced by equal servings of whole grains, fruits or vegetables, we observed lower incidence of CRC with multivariable HR of 0.85 (95% CI, 0.77, 0.94), 0.88 (95% CI, 0.80, 0.98) or 0.89 (95% CI, 0.82, 0.98), respectively (Figure 2C). The associations for hPDI and uPDI were attenuated after adjusting for whole grains and refined grains, respectively (Supplementary Table 5).

Among all incident CRC cases, 1244 cases had available data on tumour molecular subtypes. Patients with available molecular marker data generally had similar characteristics to those without molecular data (Supplementary Table 7). No significant heterogeneity by molecular marker data availability was observed for the association of hPDI (or uPDI) and CRC (Supplementary Table 8). We integrated the IPW method into the Cox regression models for subsequent analyses to adjust for potential selection bias due to varied molecular data availability. The association of hPDI and CRC incidence significantly differed by *KRAS* mutation status (*P*‐heterogeneity = .003) (Table 3). A higher hPDI was associated with lower incidence of *KRAS*‐wildtype CRC (multivariable HR comparing extreme quartiles, 0.74, 95% CI: 0.57, 0.96; *P*‐trend = .004) but not *KRAS*‐mutant CRC (multivariable HR comparing extreme quartiles, 1.10, 95% CI: 0.82, 1.47; *P*‐trend = .22). We did not observe evidence of heterogeneity by MSI, CIMP or *BRAF* status for hPDI (Table 3) or by any of the four molecular markers for uPDI (Supplementary Table 9) (*P*‐heterogeneity ≥ .15). When defining CRC molecular subtypes using the four molecular markers in combination,^38^ the association of hPDI and CRC was mainly observed for Type 4 CRC (non‐MSI‐high, CIMP‐low/negative, *BRAF *wild‐type, *KRAS *wild‐type) (Supplementary Table 10). The results were generally similar when analysing each cohort separately (Supplementary Tables 11 and 12). Sensitivity analyses using Cox regression models without IPW also generated similar results (Supplementary Table 13). Further analyses within the colon and rectum indicated that the differential associations of hPDI and CRC by *KRAS* mutation status was mainly for the colon cancer (Supplementary Table 14). In the analysis of individual plant food group with CRC by *KRAS* mutation status, the association of whole grains with CRC differed by *KRAS* mutation status (*P*‐heterogeneity = .05), although statistical significance was unattained at the predefined α level of 0.005 (Supplementary Table 15).

**TABLE 3:** Hazard ratios with 95% confidence intervals of incident colorectal cancer (CRC) subclassified by tumour molecular features according to the healthy plant‐based diet index in the pooled cohort^a^

	Quartiles of healthy plant‐based diet index					
	Quartile 1	Quartile 2	Quartile 3	Quartile 4	*P*‐trend^b^	*P*‐heterogeneity^c^
**MSI status**						.84
Non‐MSI‐high CRC						
No. of cases	231	256	272	242		
Age‐adjusted	1 (reference)	1.11 (0.91, 1.35)	1.02 (0.84, 1.24)	0.86 (0.70, 1.05)	.05	
Multivariable‐adjusted	1 (reference)	1.12 (0.92, 1.36)	1.03 (0.84, 1.26)	0.88 (0.71, 1.09)	.14	
MSI‐high CRC						
No. of cases	48	40	52	50		
Age‐adjusted	1 (reference)	0.58 (0.38, 0.90)	0.79 (0.52, 1.19)	0.72 (0.47, 1.10)	.30	
Multivariable‐adjusted	1 (reference)	0.58 (0.38, 0.90)	0.79 (0.52, 1.20)	0.75 (0.49, 1.15)	.39	
**CIMP status**						.90
CIMP‐low/negative CRC						
No. of cases	211	243	270	236		
Age‐adjusted	1 (reference)	1.13 (0.92, 1.39)	1.08 (0.88, 1.32)	0.92 (0.75, 1.13)	.16	
Multivariable‐adjusted	1 (reference)	1.15 (0.94, 1.41)	1.11 (0.90, 1.36)	0.96 (0.78, 1.20)	.41	
CIMP‐high CRC						
No. of cases	53	39	52	56		
Age‐adjusted	1 (reference)	0.58 (0.38, 0.89)	0.81 (0.53, 1.23)	0.77 (0.52, 1.16)	.56	
Multivariable‐adjusted	1 (reference)	0.58 (0.38, 0.88)	0.83 (0.54, 1.26)	0.82 (0.55, 1.24)	.80	
***BRAF* mutation status**						.22
*BRAF*‐wildtype CRC						
No. of cases	231	257	285	256		
Age‐adjusted	1 (reference)	1.11 (0.91, 1.35)	1.07 (0.88, 1.30)	0.90 (0.74, 1.09)	.21	
Multivariable‐adjusted	1 (reference)	1.11 (0.92, 1.36)	1.08 (0.89, 1.32)	0.92 (0.75, 1.14)	.24	
*BRAF*‐mutant CRC						
No. of cases	49	40	48	41		
Age‐adjusted	1 (reference)	0.68 (0.43, 1.06)	0.75 (0.48, 1.17)	0.61 (0.39, 0.96)	.10	
Multivariable‐adjusted	1 (reference)	0.67 (0.43, 1.05)	0.75 (0.48, 1.17)	0.63 (0.40, 1.00)	.06	
***KRAS* mutation status**						.003
*KRAS*‐wildtype CRC						
No. of cases	161	181	169	149		
Age‐adjusted	1 (reference)	1.02 (0.81, 1.29)	0.85 (0.67, 1.07)	0.69 (0.54, 0.89)	<.001	
Multivariable‐adjusted	1 (reference)	1.03 (0.82, 1.31)	0.87 (0.68, 1.11)	0.74 (0.57, 0.96)	.004	
*KRAS*‐mutant CRC						
No. of cases	106	101	144	133		
Age‐adjusted	1 (reference)	1.00 (0.74, 1.35)	1.16 (0.88, 1.54)	1.02 (0.77, 1.36)	.47	
Multivariable‐adjusted	1 (reference)	1.03 (0.76, 1.40)	1.22 (0.92, 1.62)	1.10 (0.82, 1.47)	.22	

^a^ All analyses were stratified by age (in month), calendar year and sex. Multivariable‐adjusted hazard ratios were adjusted for body mass index (continuous with a ceiling at 35 kg/m2), physical activity (continuous with a ceiling at 50 metabolic equivalent task score‐hours/week), smoking status (never, past or current), regular use of aspirin or other non‐steroidal anti‐inflammatory drugs (≥2 tablets per week: yes or no), family history of colorectal cancer (yes or no), history of previous lower gastrointestinal endoscopy (yes or no), alcohol intake (continuous with a ceiling at 30 g/day) and total energy intake (continuous). The inverse probability weighting method was applied to reduce selection bias due to molecular data availability.

^b^ The healthy plant‐based diet index was used as a continuous variable in the regression model except for individuals below 5th percentile and those above 95th percentile for whom the 5th and 95th percentile values, respectively, were used to eliminate outlier effects.

^c^ We tested for heterogeneity by using a likelihood ratio test, comparing a multivariable‐adjusted model that allows separate associations for different colorectal cancer subtypes with a model that assumes a common association.

Abbreviations: CIMP, CpG island methylator phenotype; CRC, colorectal cancer; MSI, microsatellite instability.

We conducted subgroup analyses for overall CRC by age or body mass index and did not observe any significant effect modification (Supplementary Table 16). We also conducted sensitivity analysis excluding early‐onset CRCs that were diagnosed in participants under 50 years old (*n* = 60) and confirmed that the observed associations persisted for later‐onset CRC (Supplementary Table 17). Last, the results for the association of hPDI and uPDI with overall CRC and CRC molecular subtypes remained similar in sensitivity analyses where we stopped further updating dietary data after diagnosis of other disease outcomes that might change a person's habitual diet (Supplementary Table 18 and Supplementary Table 19).

## 4 DISCUSSION

Colorectal adenocarcinomas are heterogeneous multifactorial diseases, the incidence and characteristics of which are modified by diet and lifestyle.^45^, ^46^ Our current study demonstrated an inverse association between the healthy plant‐based diet index (hPDI) and colorectal cancer (CRC) incidence. The reduced CRC incidence associated with a high hPDI was only observed for *KRAS*‐wildtype CRC but not the *KRAS*‐mutated subtype. In contrast, the unhealthy plant‐based diet index (uPDI) was associated with increased CRC incidence. Replacing refined grains with healthy plant foods such as whole grains, fruits and vegetables was associated with lower CRC incidence.

A few previous cohort studies have examined the association of plant‐derived food intake with CRC risk.^47^, ^48^, ^49^ The results were mixed, with one study showing an inverse association^49^ and others reporting null findings.^47^, ^48^ One key limitation was that the quality of the plant foods in these studies was not adequately differentiated. Unhealthy plant foods such as refined grains have been associated with a higher CRC risk.^12^ Therefore, intake of detailed plant‐based food items needs to be measured. The food consumption patterns were not described in the two studies that reported a null association between vegetarian diets and CRC risk.^47^, ^48^ However, in the study where the inverse association was observed,^49^ compared to non‐vegetarians, vegetarians consumed on average lower amounts of refined grains, sweets, snack foods and caloric beverages, in addition to reduced consumption of animal products.^50^ Such a vegetarian diet was similar to the healthy plant‐based diet in our study. Thus, the findings in that study^49^ and our current study consistently indicate a possible role of healthy plant‐based diets in CRC prevention.

Other plant‐based dietary patterns, such as the Alternative Mediterranean Diet (AMED), Dietary Approaches to Stop Hypertension (DASH) diet and prudent diet, have also been associated with a lower CRC risk.^51^ Healthy plant‐based diet is correlated to these dietary patterns (Supplementary Table 20). However, there are notable differences among these various plant‐based dietary patterns. For example, fish intake was given a positive weight in the prudent diet and AMED, and low‐fat dairy was given a positive weight in the DASH diet.^51^ Prudent diet, DASH diet and AMED no doubt reflect healthy eating habits, highlighting both healthy plant and healthy animal foods. Our study aimed to differentiate between the healthy and unhealthy plant foods and gave negative weights to all animal foods. There is increasing interest in the plant‐based diet because of its benefits to both human health and environmental sustainability. Therefore, it is important to characterise the better nature of healthy plant foods compared to both animal‐based foods and unhealthy plant foods.

Although one recent study suggested that the total protein intake underlined the health effects of plant‐based diets,^41^ our analyses of protein and fat did not show any associations between protein or fat intake and CRC incidence, suggesting other components of plant‐based diets might explain the beneficial effects. Analyses of individual food groups demonstrated that the inverse association between the healthy plant‐based diet and CRC incidence could be primarily ascribed to a higher intake of whole grains and a lower intake of refined grains. There is substantial evidence that whole grains and foods containing dietary fibre are associated with a reduced CRC risk.^3^ Whole grains are good sources of dietary fibre and may decrease the risk of CRC by increasing stool bulk and decreasing transit time, thus reducing the contact between potential carcinogens and colorectal epithelial cells.^52^ In addition, microbial fermentation of fibre produces short‐chain fatty acids, which may regulate the immune system and reduce CRC risk.^4^ Other beneficial nutritional components in whole grains, such as polyphenols, lignans and phytoestrogens, which are found mainly in the bran and germ of the grain, may also protect against CRC.^53^, ^54^ These bioactive compounds that are missing in refined grains might help explain both the existence of an inverse association between whole grains and CRC risk and a lack of association with dietary fibre in some studies, as refined grains could also be a source of dietary fibre.

We took the molecular pathological epidemiology approach in which we attempted to link the putative etiological factors (hPDI and uPDI) with specific tumour molecular signatures.^45^ We observed heterogeneity of the association between hPDI and CRC incidence by *KRAS* mutation status. A higher hPDI was associated with reduced incidence of *KRAS*‐wildtype CRC but not *KRAS*‐mutant CRC. It is well‐recognised that *KRAS* mutations in CRC confer resistance to anti‐EGFR targeted therapy.^55^, ^56^ As our current study suggests, *KRAS*‐mutated colorectal neoplasms may also be resistant to the beneficial effects of hPDI. Experimental evidence indicates that polyphenols in healthy plant foods, such as ferulic acid and p‐coumaric acid in whole grains,^57^, ^58^ hydroxytyrosol in olive oil^59^ and epigallocatechin‐3‐gallate in green tea,^60^ can inhibit colorectal tumour cell growth via downregulation of EGFR expression. The resistance of *KRAS*‐mutant tumour cells to alterations in EGFR signalling might result in the null association between hPDI and *KRAS*‐mutant CRC. An alternative explanation could be that unhealthy diets, indicated by a lower hPDI, are not responsible for the multiple *KRAS* mutations that lead to the initiation of CRC, suggesting looking for other biological mechanisms.^61^ In the analyses of CRC subtypes defined by all four molecular markers, we observed a beneficial association between hPDI and Type 4 CRC (defined by Jass^38^). These findings are consistent with a previous analysis reporting that dietary factors including total dietary fibre and total folate were generally more strongly associated with conventional non‐serrated adenomas than with serrated lesions.^62^ Although future studies are needed to elucidate the mechanisms underlying our findings, our approach can provide novel insight into the possible cancer‐preventive effects of healthy plant‐based diets.

Several strengths of our study are apparent. First, the prospective cohort design eliminated differential recall bias between individuals with and without CRC. Second, IPW method was applied to adjust for selection bias. Third, repeated assessments of diet allowed us to assess long‐term dietary habits and patterns in relation to CRC incidence. Fourth, the molecular pathological epidemiology approach^16^, ^45^, ^63^, ^64^, ^65^, ^66^ enabled us to assess the etiological link between the dietary patterns and specific molecular subtypes, thereby providing pathogenic insight into the observed epidemiological association.

Nevertheless, we acknowledge several limitations. First, as in any other observational study, there existed unmeasured and residual confounding to uncertain degrees. However, residual confounding is likely smaller compared to most previous studies, given our detailed and repeated measurement of diet and covariates. Similar results generated from several sensitivity analyses also gave us reassurance of the robustness of our findings. Second, our data on dietary intake were derived from responses of study participants to the questionnaires and had certain measurement errors. However, the FFQs used in our study were thoroughly validated against dietary records.^23^, ^24^ Third, not all incident CRC cases provided tissue materials for molecular analyses. Nonetheless, the employed IPW method enabled adjusting for potential selection bias due to the varied availability of stored carcinomatous tissue, and the results using the IPW approach were similar to those not using the IPW method. In addition, the small sample size for subgroup analyses based on tumour characteristics limited our power to detect the heterogeneity. Finally, all of our participants were health care workers, and a vast majority of them were white. Future research is necessary to examine similar hypotheses in other population groups.

In summary, our current study revealed an inverse association between a healthy plant‐based diet and the incidence of CRC, particularly the *KRAS*‐wildtype subtype, as well as a positive association between unhealthy plant‐based diet and CRC incidence. Our data not only underscore the necessity of differentiating healthy plant foods from unhealthy plant foods when advocating implementation of plant‐based diets for CRC prevention but also highlight the modifying effects of tumour characteristics on the association of diet with CRC risk.

## FUNDING

This work was supported by grants from the US National Institutes of Health (UM1 CA186107, U01 CA167552, UM1 CA167552, P01 CA87969, P01 CA55075, R01 CA151993, R35 CA197735) and by Cancer Research UK Grand Challenge Award (UK C10674/A27140 to SO). TU was supported by a grant from Overseas Research Fellowship (201960541 to TU) from Japan Society for the Promotion of Science and a grant from the Prevent Cancer Foundation. The sponsors had no role in the study design; the collection, analysis or interpretation of data; the writing of the report or in the decision to submit the article for publication.

## USE OF STANDARDISED OFFICIAL SYMBOLS

We use HUGO (Human Genome Organisation)‐approved official symbols (or root symbols) for genes and gene products, including BRAF, CACNA1G, CDKN2A, CRABP1, EGFR, IGF2, KRAS, MLH1, NEUROG1, RUNX3 and SOCS1, all of which are described at www.genenames.org. Gene symbols are italicised, whereas symbols for gene products are not italicised.

## CONFLICT OF INTEREST

ATC has served as an investigator on a separate study of personalised diet sponsored by Zoe Global Ltd. MG receives research funding from Bristol‐Myers Squibb, Merck, Servier and Janssen. JAM has received institutional research funding from Boston Biomedical, has served as an advisor/consultant to COTA Healthcare, and served on a grant review panel for the National Comprehensive Cancer Network funded by Taiho Pharmaceutical.

## Supporting information

Supporting Information
