# Supplementary material for: Healthy and unhealthy plant‐based diets in relation to the incidence of colorectal cancer overall and by molecular subtypes
Source: Clin Transl Med. 2022 Aug 23;12(8):e893. doi: 10.1002/ctm2.893 (PMC9398226; doi:10.1002/ctm2.893)
Supplement: Supplementary file 1 — Supporting Information [file CTM2-12-e893-s001.docx]

**Supplementary Tables**

**Supplementary Table 1.** Specific *BRAF* and *KRAS* mutations in this study

**Supplementary Table 2.** Age-standardized characteristics of participants in the Nurses’ Health Study and the Health Professionals Follow-up Study, according to quartiles of the unhealthy plant-based diet index

**Supplementary Table 3**. Hazard ratios with 95% confidence intervals of incident colorectal cancer by tumor location according to the healthy and unhealthy plant-based diet indices (per 15-unit, the difference between the median score values of extreme quartiles) in the separate cohorts and the pooled cohort

**Supplementary Table 4.** Hazard ratios with 95% confidence intervals of incident colorectal cancer according to the overall plant-based diet index in the Nurses’ Health Study (NHS), the Health Professionals Follow-up Study (HPFS), and the pooled cohort

**Supplementary Table 5.** Hazard ratios with 95% confidence intervals of incident colorectal cancer according to the healthy or unhealthy plant-based diet index in the pooled cohorts, adjusting for intake of fish/seafood, whole grains, and refined grains

**Supplementary Table 6.** Hazard ratios with 95% confidence intervals of incident colorectal cancer according to total protein, protein from plant source, protein from animal source, total fat, fat from plant source, and fat from animal source in the pooled cohort

**Supplementary Table 7**. Characteristics of colorectal cancer patients with and without molecular marker data by cohort

**Supplementary Table 8**. Hazard ratios with 95% confidence intervals of incident colorectal cancer according to the healthy and unhealthy plant-based diet indices (per 15-unit, the difference between the median score values of extreme quartiles) in the pooled cohort, stratified by molecular data availability

**Supplementary Table 9.** Hazard ratios with 95% confidence intervals of incident colorectal cancer (CRC) by tumor molecular features according to the unhealthy plant-based diet index in the pooled cohort

**Supplementary Table 10**. Hazard ratios (HR) and 95% confidence intervals (CI) of incident colorectal cancer by combined molecular subtypes according to the healthy and unhealthy plant-based diet indices (per 15-unit, the difference between the median score values of extreme quartiles) in the pooled cohort

**Supplementary Table 11**. Hazard ratios with 95% confidence intervals of incident colorectal cancer by tumor molecular features according to the healthy and unhealthy plant-based diet indices (per 15-unit, the difference between the median score values of extreme quartiles) in the separate cohorts

**Supplementary Table 12**. Hazard ratios (HR) and 95% confidence intervals (CI) of incident colorectal cancer by combined molecular subtypes according to the healthy and unhealthy plant-based diet indices (per 15-unit, the difference between the median score values of extreme quartiles) in the separate cohorts

**Supplementary Table 13.** Hazard ratios with 95% confidence intervals of incident colorectal cancer (CRC) by tumor molecular features according to the healthy or unhealthy plant-based diet indices in the pooled cohort, without using inverse probability weighting

**Supplementary Table 14**. Hazard ratios with 95% confidence intervals of incident colon cancer and rectal cancer by tumor molecular features according to the healthy and unhealthy plant-based diet indices (per 15-unit, the difference between the median score values of extreme quartiles) in the pooled cohort

**Supplementary Table 15**. Multivariable hazard ratios with 95% confidence intervals of incident colorectal cancer subclassified by *KRAS* mutation status according to individual plant food (per one serving/day) in the pooled cohort

**Supplementary Table 16**. Hazard ratios with 95% confidence intervals of incident colorectal cancer according to the healthy and unhealthy plant-based diet indices (per 15-unit, the difference between the median score values of extreme quartiles) in the pooled cohort, stratified by age and body mass index

**Supplementary Table 17**. Hazard ratios with 95% confidence intervals of incident later-onset colorectal cancer (diagnosed before age 50) according to the healthy and unhealthy plant-based diet indices in the pooled cohort

**Supplementary Table 18.** Hazard ratios with 95% confidence intervals of incident colorectal cancer according to the healthy or unhealthy plant-based diet index in the Nurses’ Health Study (NHS) and Health Professionals Follow-up Study (HPFS), stopped updating diet after the diagnosis of other disease outcomes

**Supplementary Table 19.** Hazard ratios with 95% confidence intervals of incident colorectal cancer (CRC) by tumor molecular features according to the healthy or unhealthy plant-based diet indices in the pooled cohort, stopped updating diet after the diagnosis of other disease outcomes

**Supplementary Table 20**. Pearson correlations between plant-based diet indices and other dietary indices in the pooled cohorts

**Supplementary Figures**

**Supplementary Figure 1.** Distribution of the three plant-based diet indices in the Nurses’ Health Study and the Health Professionals Follow-up Study

**Supplementary Figure 2**. Average values of the three plant-based diet indices in the two cohorts across the follow-up period

Supplementary Table 1. Specific *BRAF* and *KRAS* mutations in this study

| **Codon** | **Nucleotide change** | **Amino acid change** | **Codon change** |
| --- | --- | --- | --- |
| *BRAF* | | | |
| 600 | c.1799T>A | p.V600E | GTG>GAG |
| *KRAS* | | | |
| 12 | c.34G>A | p.G12S | GGT>AGT |
| 12 | c.34G>C | p.G12R | GGT>CGT |
| 12 | c.34G>T | p.G12C | GGT>TGT |
| 12 | c.35G>A | p.G12D | GGT>GAT |
| 12 | c.35G>C | p.G12A | GGT>GCT |
| 12 | c.35G>T | p.G12V | GGT>GTT |
| 12 | c.35_36delinsCA | p.G12A | GGT>GCA |
| 13 | c.37G>C | p.G13R | GGC>CGC |
| 13 | c.37G>T | p.G13C | GGC>TGC |
| 13 | c.38G>A | p.G13D | GGC>GAC |
| 13 | c.38G>T | p.G13V | GGC>GTC |
| 61 | c.182A>G | p.Q61R | CAA>CGA |
| 61 | c.182A>T | p.Q61L | CAA>CTA |
| 61 | c.183A>C | p.Q61H | CAA>CAC |
| 60, 61 | c.180_181delinsAA | p.Q61K | GGT + CAA>GGA + AAA |
| 146 | c.436G>A | p.A146T | GCA>ACA |
| 146 | c.436G>C | p.A146P | GCA>CCA |
| 146 | c.437C>T | p.A146V | GCA>GTA |
| 12, 13 | c.35G>A, c.38G>A | p.G12D, p.G13D | GGT>GAT, GGC>GAC |
| 12, 13 | c.35G>T, c.37G>T | p.G12V, p.G13C | GGT>GTT, GGC>TGC |
| 12,  60, 61 | c.35G>T, c.180_181delinsAA | p.G12V,  p.Q61K | GGT>GTT, GGT + CAA>GGA + AAA |
| 12, 146 | c.34G>C, c.436G>A | p.G12R, p.A146T | GGT>CGT, GCA>ACA |
| 12, 146 | c.34G>T, c.436G>A | p.G12C, p.A146T | GGT>TGT, GCA>ACA |
| 12, 146 | c.34G>T, c.437C>T | p.G12C, p.A146V | GGT>TGT, GCA>GTA |
| 12, 146 | c.35G>T, c.436G>A | p.G12V, p.A146T | GGT>GTT, GCA>ACA |
| 13, 146 | c.38G>A, c.436G>A | p.G13D, p.A146T | GGC>GAC, GCA>ACA |
| 12, 13,  61 | c.35G>A, c.38G>A, c.183A>T | p.G12D, p.G13D,  p.Q61H | GGT>GAT, GGC>GAC, CAA>CAT |

Supplementary Table 2. Age-standardized characteristics of participants in the Nurses’ Health Study and the Health Professionals Follow-up Study, according to quartiles of the unhealthy plant-based diet index

|  | Nurses’ Health Study | | | |  | Health Professionals Follow-up Study | | | |
| --- | --- | --- | --- | --- | --- | --- | --- | --- | --- |
|  | Quartile 1 | Quartile 2 | Quartile 3 | Quartile 4 |  | Quartile 1 | Quartile 2 | Quartile 3 | Quartile 4 |
| Person-years | 513,790 | 513,308 | 509,437 | 515,253 |  | 269,837 | 272,645 | 277,121 | 271,765 |
| Age at baseline, years (mean) | 50 | 50 | 50 | 49 |  | 54 | 53 | 52 | 52 |
| Body mass index, kg/m^2^ (mean) | 26.1 | 25.8 | 25.7 | 25.5 |  | 25.8 | 25.5 | 25.4 | 25.2 |
| Physical activity, METS-hour/week (mean) | 20.4 | 17.3 | 15.2 | 12.6 |  | 35.7 | 32.4 | 29.6 | 25.6 |
| Current smoker (%) | 10 | 12 | 14 | 15 |  | 6 | 6 | 7 | 6 |
| Non-drinker of alcohol (%) | 18 | 21 | 24 | 32 |  | 13 | 15 | 18 | 25 |
| History of previous endoscopy (%) | 24 | 23 | 23 | 21 |  | 35 | 35 | 34 | 33 |
| Family history of colorectal cancer (%) | 20 | 20 | 19 | 19 |  | 15 | 15 | 15 | 14 |
| Regular use of aspirin or other nonsteroidal anti-inflammatory drugs (%) | 35 | 33 | 33 | 32 |  | 38 | 38 | 36 | 35 |
| Premenopausal (%) | 11 | 12 | 12 | 12 |  | / | / | / | / |
| Current postmenopausal hormone use (%) | 28 | 26 | 25 | 23 |  | / | / | / | / |
| Dietary intake (mean) |  |  |  |  |  |  |  |  |  |
| Alcohol, among drinkers, g/day | 8.6 | 8.1 | 7.6 | 6.8 |  | 15.4 | 13.7 | 13.0 | 11.1 |
| Total energy, kcal/day | 1967 | 1788 | 1669 | 1540 |  | 2259 | 2028 | 1886 | 1737 |
| Total dietary fiber, g/day | 20.2 | 18.5 | 17.2 | 15.3 |  | 24.7 | 23.1 | 21.5 | 19.1 |
| Total folate, µg/day | 505 | 471 | 443 | 408 |  | 599 | 560 | 536 | 498 |
| Healthy plant foods |  |  |  |  |  |  |  |  |  |
| Whole grains, serving/day | 1.8 | 1.4 | 1.2 | 0.8 |  | 2.2 | 1.8 | 1.5 | 1.1 |
| Fruits, serving/day | 2.2 | 1.7 | 1.4 | 1.0 |  | 2.3 | 1.8 | 1.5 | 1.1 |
| Vegetables, serving/day | 4.4 | 3.4 | 2.8 | 2.0 |  | 4.3 | 3.4 | 2.8 | 2.0 |
| Legumes, serving/week | 3.7 | 3.0 | 2.5 | 2.0 |  | 4.2 | 3.4 | 2.8 | 2.2 |
| Nuts, serving/week | 2.3 | 1.8 | 1.4 | 1.1 |  | 3.7 | 2.8 | 2.2 | 1.7 |
| Vegetable oils, serving/week | 3.4 | 2.4 | 1.7 | 1.0 |  | 3.2 | 2.3 | 1.8 | 1.2 |
| Tea/coffee, serving/day | 3.4 | 3.0 | 2.8 | 2.4 |  | 2.8 | 2.4 | 2.2 | 1.7 |
| Unhealthy plant foods |  |  |  |  |  |  |  |  |  |
| Refined grains, serving/day | 1.5 | 1.6 | 1.6 | 1.7 |  | 1.5 | 1.5 | 1.6 | 1.7 |
| Sweets/desserts, serving/day | 1.1 | 1.2 | 1.3 | 1.4 |  | 1.2 | 1.4 | 1.4 | 1.6 |
| Potatoes, serving/week | 3.1 | 3.3 | 3.4 | 3.7 |  | 3.7 | 3.8 | 3.8 | 3.9 |
| Fruit juice, serving/week | 4.9 | 5.2 | 5.1 | 5.1 |  | 5.2 | 5.4 | 5.5 | 5.8 |
| Sugar-sweetened beverages,  serving/week | 0.9 | 1.4 | 2.0 | 3.1 |  | 1.4 | 2.0 | 2.5 | 3.6 |
| Animal foods |  |  |  |  |  |  |  |  |  |
| Animal fats, serving/week | 2.9 | 2.4 | 2.1 | 1.8 |  | 2.7 | 1.8 | 1.5 | 1.2 |
| Dairy products, serving/day | 2.5 | 2.2 | 1.9 | 1.6 |  | 2.4 | 2.0 | 1.8 | 1.5 |
| Eggs, serving/week | 2.6 | 2.1 | 1.8 | 1.4 |  | 2.7 | 2.1 | 1.8 | 1.5 |
| Fish/seafood, serving/week | 3.2 | 2.4 | 1.9 | 1.4 |  | 3.5 | 2.8 | 2.4 | 1.8 |
| Meat, serving/day | 1.7 | 1.6 | 1.5 | 1.4 |  | 2.0 | 1.8 | 1.6 | 1.5 |
| Miscellaneous animal foods,  serving/week | 3.4 | 2.9 | 2.6 | 2.1 |  | 3.4 | 2.8 | 2.5 | 2.0 |
| Healthy plant-based diet index (mean) | 57.8 | 55.7 | 54.2 | 52.0 |  | 57.4 | 55.8 | 54.3 | 52.0 |
| Unhealthy plant-based diet index (mean) | 46.2 | 52.5 | 57.0 | 63.3 |  | 46.5 | 52.5 | 56.8 | 62.7 |

All variables are standardized to the age distribution of the study population, except for age at baseline.

Abbreviations: METS, metabolic equivalent task score.

Supplementary Table 3. Hazard ratios with 95% confidence intervals of incident colorectal cancer by tumor location according to the healthy and unhealthy plant-based diet indices (per 15-unit, the difference between the median score values of extreme quartiles) in the separate cohorts and the pooled cohort^a^

|  | Healthy plant-based diet index | Unhealthy plant-based diet index |
| --- | --- | --- |
|  | Nurses’ Health Study | |
| **Proximal colon cancer (n=827)** |  |  |
| Multivariable-adjusted | 0.92 (0.75, 1.11) | 1.21 (1.01, 1.45) |
| **Distal colon cancer (n=458)** |  |  |
| Multivariable-adjusted | 0.94 (0.73, 1.20) | 0.97 (0.77, 1.23) |
| **Rectal cancer (n=349)** |  |  |
| Multivariable-adjusted | 0.89 (0.66, 1.18) | 1.22 (0.93, 1.59) |
| P heterogeneity^b^ | 0.96 | 0.27 |
|  | Health Professionals Follow-up Study | |
| **Proximal colon cancer (n=477)** |  |  |
| Multivariable-adjusted | 0.83 (0.65, 1.05) | 1.05 (0.82, 1.34) |
| **Distal colon cancer (n=395)** |  |  |
| Multivariable-adjusted | 0.91 (0.70, 1.17) | 1.21 (0.93, 1.58) |
| **Rectal cancer (n=287)** |  |  |
| Multivariable-adjusted | 1.05 (0.78, 1.41) | 1.07 (0.79, 1.46) |
| P heterogeneity^b^ | 0.47 | 0.71 |
|  | Pooled cohort | |
| **Proximal colon cancer (n=1304)** |  |  |
| Multivariable-adjusted | 0.88 (0.75, 1.03) | 1.21 (1.03, 1.40) |
| **Distal colon cancer (n=853)** |  |  |
| Multivariable-adjusted | 0.93 (0.77, 1.13) | 1.03 (0.86, 1.24) |
| **Rectal cancer (n=636)** |  |  |
| Multivariable-adjusted | 0.94 (0.75, 1.17) | 1.14 (0.91, 1.41) |
| P heterogeneity^b^ | 0.85 | 0.45 |

^a^All analyses were stratified by age (in month), calendar year, and sex. Multivariable-adjusted hazard ratios were adjusted for body mass index (continuous with a ceiling at 35 kg/m^2^), physical activity (continuous with a ceiling at 50 metabolic equivalent task score-hours/week), smoking status (never, past, or current), regular use of aspirin or other non-steroidal anti-inflammatory drugs (≥2 tablets per week: yes or no), family history of colorectal cancer (yes or no), history of previous lower gastrointestinal endoscopy (yes or no), alcohol intake (continuous with a ceiling at 30 g/day), and total energy intake (continuous) .

^b^We tested for heterogeneity by using a likelihood ratio test, comparing a multivariable-adjusted model that allows separate associations for the three colorectal cancer by tumor location with a model that assumes a common association.

Supplementary Table 4. Hazard ratios with 95% confidence intervals of incident colorectal cancer according to the overall plant-based diet index in the Nurses’ Health Study (NHS), the Health Professionals Follow-up Study (HPFS), and the pooled cohort^a^

|  | Quartiles of overall plant-based diet index | | | | P-trend^b^ |
| --- | --- | --- | --- | --- | --- |
|  | Quartile 1 | Quartile 2 | Quartile 3 | Quartile 4 |  |
| **NHS** |  |  |  |  |  |
| No. of cases | 393 | 437 | 432 | 426 |  |
| Age-adjusted | 1 (reference) | 1.01 (0.88, 1.16) | 1.00 (0.87, 1.14) | 0.92 (0.80, 1.06) | 0.32 |
| Multivariable-adjusted | 1 (reference) | 1.06 (0.92, 1.22) | 1.07 (0.93, 1.24) | 1.02 (0.87, 1.19) | 0.59 |
| **HPFS** |  |  |  |  |  |
| No. of cases | 348 | 330 | 369 | 342 |  |
| Age-adjusted | 1 (reference) | 0.89 (0.76, 1.03) | 0.94 (0.81, 1.09) | 0.88 (0.76, 1.03) | 0.08 |
| Multivariable-adjusted | 1 (reference) | 0.94 (0.80, 1.09) | 1.03 (0.88, 1.20) | 1.02 (0.86, 1.20) | 0.87 |
| **Pooled** |  |  |  |  |  |
| No. of cases | 741 | 767 | 801 | 768 |  |
| Age-adjusted | 1 (reference) | 0.95 (0.86, 1.06) | 0.97 (0.88, 1.07) | 0.90 (0.82, 1.00) | 0.05 |
| Multivariable-adjusted | 1 (reference) | 1.00 (0.90, 1.11) | 1.05 (0.95, 1.17) | 1.02 (0.91, 1.14) | 0.59 |

^a^All analyses were stratified by age (in month), calendar year, and sex. Multivariable-adjusted hazard ratios were adjusted for body mass index (continuous with a ceiling at 35 kg/m^2^), physical activity (continuous with a ceiling at 50 metabolic equivalent task score-hours/week), smoking status (never, past, or current), regular use of aspirin or other non-steroidal anti-inflammatory drugs (≥2 tablets per week: yes or no), family history of colorectal cancer (yes or no), history of previous lower gastrointestinal endoscopy (yes or no), alcohol intake (continuous with a ceiling at 30 g/day), and total energy intake (continuous) . In NHS-only analyses, we also adjusted for postmenopausal hormone use (premenopausal, postmenopausal never, past, or current use).

^b^The overall plant-based diet index was used as a continuous variable in the regression model except for individuals below 5th percentile and those above 95th percentile for whom the 5th and 95th percentile values, respectively, were used to eliminate outlier effects.

Abbreviations: HPFS, Health Professionals Follow-up Study, NHS, Nurses’ Health Study.

Supplementary Table 5. Hazard ratios with 95% confidence intervals of incident colorectal cancer according to the healthy or unhealthy plant-based diet index in the pooled cohorts, adjusting for intake of fish/seafood, whole grains, and refined grains

|  | Quartiles of healthy or unhealthy plant-based diet index | | | | P-trend^e^ |
| --- | --- | --- | --- | --- | --- |
|  | Quartile 1 | Quartile 2 | Quartile 3 | Quartile 4 |  |
| **Healthy plant-based diet index** | | | | | |
| Multivariable-adjusted^a^ | 1 (reference) | 0.88 (0.79, 0.97) | 0.93 (0.83, 1.03) | 0.86 (0.77, 0.96) | 0.04 |
| + fish/seafood^b^ | 1 (reference) | 0.88 (0.79, 0.98) | 0.93 (0.84, 1.04) | 0.87 (0.78, 0.97) | 0.06 |
| + whole grains^c^ | 1 (reference) | 0.90 (0.81, 1.00) | 0.97 (0.87, 1.08) | 0.92 (0.82, 1.04) | 0.61 |
| **Unhealthy plant-based diet index** | | | | | |
| Multivariable-adjusted^a^ | 1 (reference) | 1.07 (0.96, 1.18) | 1.08 (0.97, 1.20) | 1.16 (1.04, 1.29) | 0.005 |
| + fish/seafood^b^ | 1 (reference) | 1.06 (0.96, 1.18) | 1.07 (0.96, 1.19) | 1.14 (1.02, 1.29) | 0.02 |
| + refined grains^d^ | 1 (reference) | 1.05 (0.95, 1.17) | 1.05 (0.95, 1.18) | 1.12 (1.00, 1.25) | 0.04 |

^a^The multivariable models were stratified by age (in month), calendar year, and sex. Multivariable-adjusted hazard ratios were adjusted for body mass index (continuous with a ceiling at 35 kg/m^2^), physical activity (continuous with a ceiling at 50 metabolic equivalent task score-hours/week), smoking status (never, past, or current), regular use of aspirin or other non-steroidal anti-inflammatory drugs (≥2 tablets per week: yes or no), family history of colorectal cancer (yes or no), history of previous lower gastrointestinal endoscopy (yes or no), alcohol intake (continuous with a ceiling at 30 g/day), and total energy intake (continuous) . In NHS-only analyses, we also adjusted for postmenopausal hormone use (premenopausal, postmenopausal never, past, or current use).

^b^Further adjusted for fish/seafood intake.

^c^Further adjusted for whole grains intake.

^d^Further adjusted for refined grains intake.

^e^The healthy (or unhealthy) plant-based diet index was used as a continuous variable in the regression model except for individuals below 5th percentile and those above 95th percentile for whom the 5th and 95th percentile values, respectively, were used to eliminate outlier effects.

Supplementary Table 6. Hazard ratios with 95% confidence intervals of incident colorectal cancer according to total protein, protein from plant source, protein from animal source, total fat, fat from plant source, and fat from animal source in the pooled cohort^a^

|  | Quartile 1 | Quartile 2 | Quartile 3 | Quartile 4 | P-trend^b^ |
| --- | --- | --- | --- | --- | --- |
| **Total protein** |  |  |  |  |  |
| Age-adjusted | 1 (reference) | 0.93 (0.84, 1.03) | 0.92 (0.83, 1.01) | 0.88 (0.80, 0.98) | <0.001 |
| Multivariable-adjusted | 1 (reference) | 0.98 (0.88, 1.08) | 0.99 (0.89, 1.10) | 0.96 (0.86, 1.08) | 0.16 |
| **Protein from plant source** |  |  |  |  |  |
| Age-adjusted | 1 (reference) | 0.99 (0.90, 1.10) | 0.99 (0.89, 1.10) | 0.88 (0.78, 0.98) | <0.001 |
| Multivariable-adjusted | 1 (reference) | 1.07 (0.96, 1.19) | 1.12 (0.99, 1.25) | 1.07 (0.93, 1.23) | 0.95 |
| **Protein from animal source** |  |  |  |  |  |
| Age-adjusted | 1 (reference) | 0.94 (0.85, 1.04) | 0.96 (0.87, 1.07) | 0.86 (0.78, 0.96) | <0.001 |
| Multivariable-adjusted | 1 (reference) | 0.97 (0.88, 1.07) | 1.01 (0.91, 1.12) | 0.93 (0.83, 1.04) | 0.09 |
| **Total fat** |  |  |  |  |  |
| Age-adjusted | 1 (reference) | 1.15 (1.04, 1.28) | 1.14 (1.03, 1.27) | 1.19 (1.08, 1.32) | 0.007 |
| Multivariable-adjusted | 1 (reference) | 1.10 (0.99, 1.22) | 1.06 (0.95, 1.19) | 1.06 (0.94, 1.19) | 0.91 |
| **Fat from plant source** |  |  |  |  |  |
| Age-adjusted | 1 (reference) | 1.00 (0.90, 1.10) | 0.96 (0.86, 1.06) | 0.95 (0.86, 1.06) | 0.32 |
| Multivariable-adjusted | 1 (reference) | 1.01 (0.91, 1.11) | 0.97 (0.87, 1.07) | 0.96 (0.86, 1.07) | 0.39 |
| **Fat from animal source** |  |  |  |  |  |
| Age-adjusted | 1 (reference) | 1.10 (1.00, 1.22) | 1.15 (1.03, 1.27) | 1.23 (1.11, 1.36) | <0.001 |
| Multivariable-adjusted | 1 (reference) | 1.03 (0.92, 1.14) | 1.02 (0.91, 1.14) | 1.04 (0.92, 1.17) | 0.62 |

^a^All analyses were stratified by age (in month), calendar year, and sex. Multivariable-adjusted hazard ratios were adjusted for body mass index (continuous with a ceiling at 35 kg/m^2^), physical activity (continuous with a ceiling at 50 metabolic equivalent task score-hours/week), smoking status (never, past, or current), regular use of aspirin or other non-steroidal anti-inflammatory drugs (≥2 tablets per week: yes or no), family history of colorectal cancer (yes or no), history of previous lower gastrointestinal endoscopy (yes or no), alcohol intake (continuous with a ceiling at 30 g/day), intakes of total energy, total fiber, total vitamin D, total calcium, and total folate (all continuous). As previous analyses of macronutrients in our cohorts, we adjusted for fat intake when analyzing protein and adjusted for protein when analyzing fat. Thus, all estimates for protein or fat bear the meaning of isocalorically replacing with total carbohydrates.

^b^The protein or fat variable was used as a continuous variable in the regression model except for individuals below 5th percentile and those above 95th percentile for whom the 5th and 95th percentile values, respectively, were used to eliminate outlier effects.

Supplementary Table 7. Characteristics of colorectal cancer patients with and without molecular marker data by cohort

|  | Nurses’ Health Study | | |  | Health Professionals Follow-up Study | | |
| --- | --- | --- | --- | --- | --- | --- | --- |
|  | Without molecular marker data (n=1051) | With molecular marker data (n=637) | P value |  | Without molecular marker data (n=782) | With molecular marker data (n=607) | P value |
| Age at diagnosis, years (mean) | 70 | 68 | <0.001 |  | 71 | 71 | 0.27 |
| Body mass index, kg/m^2^ (mean) | 26.2 | 26.3 | 0.83 |  | 26.0 | 26.0 | 0.93 |
| Physical activity, METS-hour/week (mean) | 15.6 | 14.8 | 0.26 |  | 28.1 | 29.1 | 0.47 |
| Current smoker (%) | 11.0 | 12.6 | 0.34 |  | 6.3 | 6.9 | 0.63 |
| Non-drinkers (%) | 22.9 | 19.8 | 0.13 |  | 17.0 | 14.3 | 0.18 |
| History of previous endoscopy (%) | 17.4 | 16.4 | 0.56 |  | 25.7 | 22.2 | 0.14 |
| Family history of colorectal cancer (%) | 25.1 | 26.4 | 0.57 |  | 19.8 | 24.4 | 0.04 |
| Regular aspirin or other non-steroidal anti-inflammatory drugs use (%) | 26.1 | 28.9 | 0.21 |  | 32.2 | 38.4 | 0.02 |
| AJCC disease stage (%) |  |  | <0.001 |  |  |  | <0.001 |
| Stage I | 21.4 | 22.8 |  |  | 22.0 | 31.6 |  |
| Stage II | 21.2 | 33.1 |  |  | 12.1 | 25.5 |  |
| Stage III | 22.2 | 26.8 |  |  | 13.0 | 24.7 |  |
| Stage IV | 17.6 | 12.9 |  |  | 17.0 | 11.9 |  |
| Missing | 17.6 | 4.4 |  |  | 35.8 | 6.3 |  |
| Dietary intake (mean) |  |  |  |  |  |  |  |
| Alcohol, among drinkers, g/day | 8.8 | 7.4 | 0.02 |  | 15.3 | 16.5 | 0.18 |
| Total energy, kcal/day | 1725 | 1721 | 0.86 |  | 1952 | 1989 | 0.22 |
| Total dietary fiber, g/day | 18 | 18 | 0.66 |  | 22 | 22 | 0.37 |
| Total folate, mcg/day | 453 | 448 | 0.65 |  | 545 | 521 | 0.06 |
| Healthy plant foods, serving/day | 9.9 | 9.9 | 0.98 |  | 9.8 | 9.9 | 0.49 |
| Unhealthy plant foods, serving/day | 4.3 | 4.4 | 0.29 |  | 4.5 | 4.7 | 0.09 |
| Animal foods, serving/day | 4.8 | 4.8 | 0.86 |  | 4.9 | 4.9 | 0.96 |

Abbreviations: AJCC, American Joint Committee on Cancer; METS, metabolic equivalent task score.

Supplementary Table 8. Hazard ratios with 95% confidence intervals of incident colorectal cancer according to the healthy and unhealthy plant-based diet indices (per 15-unit, the difference between the median score values of extreme quartiles) in the pooled cohort, stratified by molecular data availability^a^

|  | Quartiles of healthy or unhealthy plant-based diet index | | | | P-trend^b^ | P-heterogeneity^c^ |
| --- | --- | --- | --- | --- | --- | --- |
|  | Quartile 1 | Quartile 2 | Quartile 3 | Quartile 4 |  |  |
| **Healthy plant-based diet index** | | | | | | |
| **Molecular data availability** |  |  |  |  |  | 0.70 |
| Without molecular data |  |  |  |  |  |  |
| No. of cases | 448 | 408 | 481 | 496 |  |  |
| Multivariable-adjusted | 1 (reference) | 0.83 (0.72, 0.95) | 0.90 (0.79, 1.03) | 0.87 (0.76, 1.00) | 0.14 |  |
| With molecular data |  |  |  |  |  |  |
| No. of cases | 291 | 306 | 340 | 307 |  |  |
| Multivariable-adjusted | 1 (reference) | 1.00 (0.84, 1.19) | 1.00 (0.83, 1.19) | 0.87 (0.73, 1.05) | 0.10 |  |
| **Unhealthy plant-based diet index** | | | | | | |
| **Molecular data availability** |  |  |  |  |  | 0.52 |
| Without molecular data |  |  |  |  |  |  |
| No. of cases | 454 | 451 | 471 | 457 |  |  |
| Multivariable-adjusted | 1 (reference) | 1.03 (0.90, 1.17) | 1.07 (0.94, 1.22) | 1.09 (0.95, 1.25) | 0.17 |  |
| With molecular data |  |  |  |  |  |  |
| No. of cases | 304 | 320 | 301 | 319 |  |  |
| Multivariable-adjusted | 1 (reference) | 1.07 (0.90, 1.27) | 1.02 (0.85, 1.22) | 1.18 (0.99, 1.41) | 0.08 |  |

^a^All analyses were stratified by age (in month), calendar year, and sex. Multivariable-adjusted hazard ratios were adjusted for body mass index (continuous with a ceiling at 35 kg/m^2^), physical activity (continuous with a ceiling at 50 metabolic equivalent task score-hours/week), smoking status (never, past, or current), regular use of aspirin or other non-steroidal anti-inflammatory drugs (≥2 tablets per week: yes or no), family history of colorectal cancer (yes or no), history of previous lower gastrointestinal endoscopy (yes or no), alcohol intake (continuous with a ceiling at 30 g/day), and total energy intake (continuous) . In NHS-only analyses, we also adjusted for postmenopausal hormone use (premenopausal, postmenopausal never, past, or current use).

^b^The overall plant-based diet index was used as a continuous variable in the regression model except for individuals below 5th percentile and those above 95th percentile for whom the 5th and 95th percentile values, respectively, were used to eliminate outlier effects.

^c^We tested for heterogeneity by using a likelihood ratio test, comparing a multivariable-adjusted model that allows separate associations for the three colorectal cancer by tumor location with a model that assumes a common association.

Supplementary Table 9. Hazard ratios with 95% confidence intervals of incident colorectal cancer (CRC) by tumor molecular features according to the unhealthy plant-based diet index in the pooled cohort^a^

|  | | Quartiles of unhealthy plant-based diet index | | | | P-trend^b^ | P-heterogeneity^c^ |
| --- | --- | --- | --- | --- | --- | --- | --- |
|  | | Quartile 1 | Quartile 2 | Quartile 3 | Quartile 4 |  |  |
| **MSI status** |  | |  |  |  |  | 0.15 |
| Non-MSI-high CRC | |  |  |  |  |  |  |
| No. of cases | | 249 | 261 | 246 | 245 |  |  |
| Age-adjusted | | 1 (reference) | 1.03 (0.85, 1.25) | 1.03 (0.84, 1.25) | 1.09 (0.89, 1.33) | 0.35 |  |
| Multivariable-adjusted | | 1 (reference) | 1.04 (0.86, 1.26) | 1.03 (0.85, 1.26) | 1.10 (0.90, 1.35) | 0.29 |  |
| MSI-high CRC | |  |  |  |  |  |  |
| No. of cases | | 41 | 45 | 44 | 60 |  |  |
| Age-adjusted | | 1 (reference) | 1.21 (0.77, 1.93) | 1.06 (0.67, 1.68) | 1.61 (1.05, 2.45) | 0.05 |  |
| Multivariable-adjusted | | 1 (reference) | 1.21 (0.76, 1.93) | 1.06 (0.67, 1.68) | 1.63 (1.05, 2.52) | 0.05 |  |
| **CIMP status** |  | |  |  |  |  | 0.36 |
| CIMP-low/negative CRC | |  |  |  |  |  |  |
| No. of cases | | 235 | 259 | 234 | 232 |  |  |
| Age-adjusted | | 1 (reference) | 1.09 (0.89, 1.33) | 1.00 (0.81, 1.22) | 1.09 (0.89, 1.34) | 0.35 |  |
| Multivariable-adjusted | | 1 (reference) | 1.09 (0.89, 1.34) | 0.99 (0.81, 1.22) | 1.09 (0.88, 1.35) | 0.35 |  |
| CIMP-high CRC | |  |  |  |  |  |  |
| No. of cases | | 46 | 48 | 49 | 57 |  |  |
| Age-adjusted | | 1 (reference) | 1.10 (0.73, 1.67) | 1.19 (0.78, 1.93) | 1.43 (0.95, 2.17) | 0.15 |  |
| Multivariable-adjusted | | 1 (reference) | 1.08 (0.71, 1.64) | 1.18 (0.77, 1.81) | 1.42 (0.94, 2.16) | 0.15 |  |
| ***BRAF* mutation status** |  | |  |  |  |  | 0.71 |
| *BRAF*-wildtype CRC | |  |  |  |  |  |  |
| No. of cases | | 260 | 260 | 247 | 262 |  |  |
| Age-adjusted | | 1 (reference) | 1.02 (0.85, 1.24) | 0.97 (0.81, 1.18) | 1.15 (0.95, 1.39) | 0.13 |  |
| Multivariable-adjusted | | 1 (reference) | 1.04 (0.86, 1.26) | 0.99 (0.81, 1.20) | 1.18 (0.97, 1.44) | 0.08 |  |
| *BRAF*-mutant CRC | |  |  |  |  |  |  |
| No. of cases | | 37 | 46 | 45 | 50 |  |  |
| Age-adjusted | | 1 (reference) | 1.14 (0.70, 1.85) | 1.25 (0.76, 2.05) | 1.29 (0.81, 2.06) | 0.27 |  |
| Multivariable-adjusted | | 1 (reference) | 1.14 (0.70, 1.85) | 1.26 (0.77, 2.05) | 1.31 (0.82, 2.08) | 0.22 |  |
| ***KRAS* mutation status** |  | |  |  |  |  | 0.45 |
| *KRAS*-wildtype CRC | |  |  |  |  |  |  |
| No. of cases | | 170 | 171 | 147 | 172 |  |  |
| Age-adjusted | | 1 (reference) | 1.04 (0.82, 1.33) | 0.88 (0.69, 1.12) | 1.09 (0.86, 1.38) | 0.66 |  |
| Multivariable-adjusted | | 1 (reference) | 1.07 (0.84, 1.36) | 0.91 (0.71, 1.16) | 1.13 (0.90, 1.43) | 0.42 |  |
| *KRAS*-mutant CRC | |  |  |  |  |  |  |
| No. of cases | | 114 | 124 | 126 | 120 |  |  |
| Age-adjusted | | 1 (reference) | 1.02 (0.77, 1.34) | 1.11 (0.84, 1.47) | 1.19 (0.89, 1.58) | 0.17 |  |
| Multivariable-adjusted | | 1 (reference) | 1.04 (0.79, 1.37) | 1.14 (0.86, 1.51) | 1.23 (0.91, 1.65) | 0.10 |  |

^a^All analyses were stratified by age (in month), calendar year, and sex. Multivariable-adjusted hazard ratios were adjusted for body mass index (continuous with a ceiling at 35 kg/m^2^), physical activity (continuous with a ceiling at 50 metabolic equivalent task score-hours/week), smoking status (never, past, or current), regular use of aspirin or other non-steroidal anti-inflammatory drugs (≥2 tablets per week: yes or no), family history of colorectal cancer (yes or no), history of previous lower gastrointestinal endoscopy (yes or no), alcohol intake (continuous with a ceiling at 30 g/day), total energy intake (continuous), and intake of six animal food groups (continuous). The inverse probability weighting method was applied to reduce selection bias due to molecular data availability.

^b^The unhealthy plant-based diet index was used as a continuous variable in the regression model except for individuals below 5th percentile and those above 95th percentile for whom the 5th and 95th percentile values, respectively, were used to eliminate outlier effects.

^c^We tested for heterogeneity by using a likelihood ratio test, comparing a multivariable-adjusted model that allows separate associations for different colorectal cancer subtypes with a model that assumes a common association.

Abbreviations: CIMP, CpG island methylator phenotype; CRC, colorectal cancer; MSI, microsatellite instability

Supplementary Table 10. Hazard ratios (HR) and 95% confidence intervals (CI) of incident colorectal cancer by combined molecular subtypes according to the healthy and unhealthy plant-based diet indices (per 15-unit, the difference between the median score values of extreme quartiles) in the pooled cohort

|  | Healthy plant-based diet index | Unhealthy plant-based diet index |
| --- | --- | --- |
| **Type 1 (n=78)** |  |  |
| Age-adjusted | 0.74 (0.40, 1.37) | 1.22 (0.67, 2.22) |
| Multivariable-adjusted | 0.80 (0.43, 1.50) | 1.23 (0.67, 1.25) |
| **Type 2 (n=26)** |  |  |
| Age-adjusted | 1.19 (0.36, 3.94) | 1.76 (0.64, 4.83) |
| Multivariable-adjusted | 1.25 (0.38, 4.16) | 1.73 (0.64, 4.67) |
| **Type 3 (n=392)** |  |  |
| Age-adjusted | 1.19 (0.91, 1.56) | 1.16 (0.88, 1.53) |
| Multivariable-adjusted | 1.27 (0.96, 1.68) | 1.18 (0.88, 1.57) |
| **Type 4 (n=396)** |  |  |
| Age-adjusted | 0.69 (0.54, 0.87) | 1.12 (0.84, 1.47) |
| Multivariable-adjusted | 0.73 (0.57, 0.93) | 1.13 (0.86, 1.49) |
| **Type 5 (n=21)** |  |  |
| Age-adjusted | 0.60 (0.12, 3.03) | 2.72 (0.85, 8.73) |
| Multivariable-adjusted | 0.62 (0.13, 3.07) | 2.82 (0.87, 9.21) |
| **Other types (n=331)** |  |  |
| Age-adjusted | 0.78 (0.58, 1.05) | 1.05 (0.76, 1.44) |
| Multivariable-adjusted | 0.84 (0.62, 1.14) | 1.07 (0.78, 1.47) |

All analyses were stratified by age (in month), calendar year, and sex. Multivariable-adjusted hazard ratios were adjusted for body mass index (continuous with a ceiling at 35 kg/m^2^), physical activity (continuous with a ceiling at 50 metabolic equivalent task score-hours/week), smoking status (never, past, or current), regular use of aspirin or other non-steroidal anti-inflammatory drugs (≥2 tablets per week: yes or no), family history of colorectal cancer (yes or no), history of previous lower gastrointestinal endoscopy (yes or no), alcohol intake (continuous with a ceiling at 30 g/day), and total energy intake (continuous) . The inverse probability weighting method was applied to reduce selection bias due to molecular data availability. Tumor subtypes described by Jass as follows: Type 1: MSI-high, CIMP-high, *BRAF* mutant, *KRAS* wild-type; Type 2: non-MSI-high, CIMP-high, *BRAF* mutant, *KRAS* wild-type; Type 3: non-MSI-high, CIMP-low/negative, *BRAF* wild-type, *KRAS* mutant; Type 4: non-MSI-high, CIMP-low/negative, *BRAF* wild-type, *KRAS* wild-type; Type 5: MSI-high, CIMP-low/negative, *BRAF* wild-type, *KRAS* wild-type.

Supplementary Table 11. Hazard ratios with 95% confidence intervals of incident colorectal cancer by tumor molecular features according to the healthy and unhealthy plant-based diet indices (per 15-unit, the difference between the median score values of extreme quartiles) in the separate cohorts

|  | Nurses’ Health Study | |  | Health Professionals Follow-up Study | |
| --- | --- | --- | --- | --- | --- |
|  | **Healthy plant-based diet index** | **Unhealthy plant-based diet index** |  | **Healthy plant-based diet index** | **Unhealthy plant-based diet index** |
| **MSI status** |  |  |  |  |  |
| Non-MSI-high |  |  |  |  |  |
| No. of cases | 486 | |  | 478 | |
| Multivariable-adjusted | 0.98 (0.76, 1.26) | 1.01 (0.80, 1.29) |  | 0.82 (0.64, 1.05) | 1.24 (0.96, 1.60) |
| MSI-high |  |  |  |  |  |
| No. of cases | 124 | |  | 66 | |
| Multivariable-adjusted | 0.78 (0.47, 1.32) | 1.37 (0.81, 2.32) |  | 1.17 (0.58, 2.36) | 1.75 (0.90, 3.42) |
| P heterogeneity | 0.43 | 0.28 |  | 0.35 | 0.33 |
| **CIMP status** |  |  |  |  |  |
| CIMP-low/negative |  |  |  |  |  |
| No. of cases | 482 | |  | 515 | |
| Multivariable-adjusted | 0.95 (0.74, 1.22) | 1.05 (0.82, 1.34) |  | 0.99 (0.77, 1.27) | 1.14 (0.87, 1.49) |
| CIMP-high |  |  |  |  |  |
| No. of cases | 134 | |  | 66 | |
| Multivariable-adjusted | 0.90 (0.57, 1.43) | 1.28 (0.79, 2.07) |  | 1.26 (0.62, 2.54) | 1.32 (0.70, 2.49) |
| P heterogeneity | 0.84 | 0.46 |  | 0.53 | 0.67 |
| ***BRAF* mutation status** |  |  |  |  |  |
| *BRAF*-wildtype |  |  |  |  |  |
| No. of cases | 488 | |  | 541 | |
| Multivariable-adjusted | 0.98 (0.76, 1.26) | 1.09 (0.85, 1.41) |  | 0.87 (0.69, 1.11) | 1.25 (0.97, 1.60) |
| *BRAF*-mutant |  |  |  |  |  |
| No. of cases | 129 | |  | 49 | |
| Multivariable-adjusted | 0.72 (0.46, 1.13) | 1.09 (0.70, 1.69) |  | 0.69 (0.32, 1.49) | 2.00 (0.97, 4.13) |
| P heterogeneity | 0.22 | 0.98 |  | 0.55 | 0.22 |
| ***KRAS* mutation status** |  |  |  |  |  |
| *KRAS*-wildtype |  |  |  |  |  |
| No. of cases | 350 | |  | 310 | |
| Multivariable-adjusted | 0.82 (0.61, 1.10) | 1.00 (0.75, 1.32) |  | 0.65 (0.48, 0.88) | 1.22 (0.90, 1.67) |
| *KRAS*-mutant |  |  |  |  |  |
| No. of cases | 225 | |  | 259 | |
| Multivariable-adjusted | 1.24 (0.86, 1.79) | 1.07 (0.74, 1.54) |  | 1.17 (0.83, 1.65) | 1.49 (1.03, 2.15) |
| P heterogeneity | 0.07 | 0.76 |  | 0.009 | 0.41 |

All analyses were stratified by age (in month), calendar year, and sex. Multivariable-adjusted hazard ratios were adjusted for body mass index (continuous with a ceiling at 35 kg/m^2^), physical activity (continuous with a ceiling at 50 metabolic equivalent task score-hours/week), smoking status (never, past, or current), regular use of aspirin or other non-steroidal anti-inflammatory drugs (≥2 tablets per week: yes or no), family history of colorectal cancer (yes or no), history of previous lower gastrointestinal endoscopy (yes or no), alcohol intake (continuous with a ceiling at 30 g/day), and total energy intake (continuous) . The inverse probability weighting method was applied to reduce selection bias due to molecular data availability.

Supplementary Table 12. Hazard ratios (HR) and 95% confidence intervals (CI) of incident colorectal cancer by combined molecular subtypes according to the healthy and unhealthy plant-based diet indices (per 15-unit, the difference between the median score values of extreme quartiles) in the separate cohorts

|  | Healthy plant-based diet index | Unhealthy plant-based diet index |
| --- | --- | --- |
|  | Nurses’ Health Study | |
| **Type 1 (n=59)** |  |  |
| Multivariable-adjusted | 0.76 (0.38, 1.56) | 0.94 (0.47, 1.88) |
| **Type 2 (n=16)** |  |  |
| Multivariable-adjusted | 1.16 (0.24, 5.65) | 2.46 (0.75, 8.08) |
| **Type 3 (n=194)** |  |  |
| Multivariable-adjusted | 1.23 (0.83, 1.83) | 1.03 (0.70, 1.52) |
| **Type 4 (n=188)** |  |  |
| Multivariable-adjusted | 0.78 (0.55, 1.10) | 1.19 (0.82, 1.73) |
| **Type 5 (n=14)** |  |  |
| Multivariable-adjusted | 0.95 (0.15, 6.21) | 2.29 (0.54, 9.69) |
| **Other types (n=166)** |  |  |
| Multivariable-adjusted | 0.88 (0.58, 1.34) | 0.97 (0.64, 1.48) |
|  | Health Professionals Follow-up Study | |
| **Type 1 (n=19)** |  |  |
| Multivariable-adjusted | 1.03 (0.30, 3.55) | 3.47 (1.34, 8.96) |
| **Type 2 (n=10)** |  |  |
| Multivariable-adjusted | 1.50 (0.38, 5.88) | 0.48 (0.12, 1.94) |
| **Type 3 (n=198)** |  |  |
| Multivariable-adjusted | 1.36 (0.93, 1.98) | 1.43 (0.95, 2.14) |
| **Type 4 (n=208)** |  |  |
| Multivariable-adjusted | 0.68 (0.50, 0.95) | 1.02 (0.70, 1.48) |
| **Type 5 (n=7)** |  |  |
| Multivariable-adjusted | 0.18 (0.02, 1.97) | 4.74 (0.90, 24.88) |
| **Other types (n=165)** |  |  |
| Multivariable-adjusted | 0.76 (0.48, 1.19) | 1.26 (0.80, 1.97) |

All analyses were stratified by age (in month), calendar year, and sex. Multivariable-adjusted hazard ratios were adjusted for body mass index (continuous with a ceiling at 35 kg/m^2^), physical activity (continuous with a ceiling at 50 metabolic equivalent task score-hours/week), smoking status (never, past, or current), regular use of aspirin or other non-steroidal anti-inflammatory drugs (≥2 tablets per week: yes or no), family history of colorectal cancer (yes or no), history of previous lower gastrointestinal endoscopy (yes or no), alcohol intake (continuous with a ceiling at 30 g/day), and total energy intake (continuous) . The inverse probability weighting method was applied to reduce selection bias due to molecular data availability. Tumor subtypes described by Jass as follows: Type 1: MSI-high, CIMP-high, *BRAF* mutant, *KRAS* wild-type; Type 2: non-MSI-high, CIMP-high, *BRAF* mutant, *KRAS* wild-type; Type 3: non-MSI-high, CIMP-low/negative, *BRAF* wild-type, *KRAS* mutant; Type 4: non-MSI-high, CIMP-low/negative, *BRAF* wild-type, *KRAS* wild-type; Type 5: MSI-high, CIMP-low/negative, *BRAF* wild-type, *KRAS* wild-type.

Supplementary Table 13. Hazard ratios with 95% confidence intervals of incident colorectal cancer (CRC) by tumor molecular features according to the healthy or unhealthy plant-based diet indices in the pooled cohort, without using inverse probability weighting^a^

|  | | Quartiles of healthy or unhealthy plant-based diet index | | | | P-trend^b^ | P-heterogeneity^c^ |
| --- | --- | --- | --- | --- | --- | --- | --- |
|  | | Quartile 1 | Quartile 2 | Quartile 3 | Quartile 4 |  |  |
| **Healthy plant-based diet index** | | | | | | | |
| **MSI status** |  | |  |  |  |  | 0.85 |
| Non-MSI-high CRC | |  |  |  |  |  |  |
| No. of cases | | 231 | 256 | 272 | 242 |  |  |
| Multivariable-adjusted | | 1 (reference) | 1.02 (0.85, 1.22) | 0.99 (0.82, 1.19) | 0.85 (0.70, 1.03) | 0.14 |  |
| MSI-high CRC | |  |  |  |  |  |  |
| No. of cases | | 48 | 40 | 52 | 50 |  |  |
| Multivariable-adjusted | | 1 (reference) | 0.70 (0.46, 1.07) | 0.84 (0.57, 1.25) | 0.75 (0.50, 1.13) | 0.40 |  |
| **CIMP status** |  | |  |  |  |  | 0.76 |
| CIMP-low/negative CRC | |  |  |  |  |  |  |
| No. of cases | | 211 | 243 | 270 | 236 |  |  |
| Multivariable-adjusted | | 1 (reference) | 1.07 (0.88, 1.29) | 1.10 (0.91, 1.33) | 0.93 (0.76, 1.14) | 0.57 |  |
| CIMP-high CRC | |  |  |  |  |  |  |
| No. of cases | | 53 | 39 | 52 | 56 |  |  |
| Multivariable-adjusted | | 1 (reference) | 0.61 (0.40, 0.92) | 0.76 (0.51, 1.12) | 0.76 (0.52, 1.12) | 0.56 |  |
| ***BRAF* mutation status** |  | |  |  |  |  | 0.21 |
| *BRAF*-wildtype CRC | |  |  |  |  |  |  |
| No. of cases | | 231 | 257 | 285 | 256 |  |  |
| Multivariable-adjusted | | 1 (reference) | 1.02 (0.85, 1.23) | 1.04 (0.87, 1.25) | 0.90 (0.74, 1.09) | 0.33 |  |
| *BRAF*-mutant CRC | |  |  |  |  |  |  |
| No. of cases | | 49 | 40 | 48 | 41 |  |  |
| Multivariable-adjusted | | 1 (reference) | 0.69 (0.45, 1.04) | 0.77 (0.52, 1.16) | 0.61 (0.40, 0.93) | 0.08 |  |
| ***KRAS* mutation status** |  | |  |  |  |  | 0.004 |
| *KRAS*-wildtype CRC | |  |  |  |  |  |  |
| No. of cases | | 161 | 181 | 169 | 149 |  |  |
| Multivariable-adjusted | | 1 (reference) | 1.01 (0.81, 1.25) | 0.89 (0.71, 1.11) | 0.74 (0.58, 0.94) | 0.006 |  |
| *KRAS*-mutant CRC | |  |  |  |  |  |  |
| No. of cases | | 106 | 101 | 144 | 133 |  |  |
| Multivariable-adjusted | | 1 (reference) | 0.90 (0.68, 1.19) | 1.14 (0.88, 1.48) | 1.04 (0.79, 1.36) | 0.22 |  |
| **Unhealthy plant-based diet index** | | | | | | | |
| **MSI status** |  | |  |  |  |  | 0.11 |
| Non-MSI-high CRC | |  |  |  |  |  |  |
| No. of cases | | 249 | 261 | 246 | 245 |  |  |
| Multivariable-adjusted | | 1 (reference) | 1.10 (0.92, 1.31) | 1.07 (0.89, 1.28) | 1.15 (0.95, 1.38) | 0.10 |  |
| MSI-high CRC | |  |  |  |  |  |  |
| No. of cases | | 41 | 45 | 44 | 60 |  |  |
| Multivariable-adjusted | | 1 (reference) | 1.15 (0.75, 1.76) | 1.14 (0.74, 1.74) | 1.66 (1.11, 2.48) | 0.02 |  |
| **CIMP status** |  | |  |  |  |  | 0.45 |
| CIMP-low/negative CRC | |  |  |  |  |  |  |
| No. of cases | | 235 | 259 | 234 | 232 |  |  |
| Multivariable-adjusted | | 1 (reference) | 1.16 (0.96, 1.38) | 1.07 (0.88, 1.29) | 1.13 (0.93, 1.37) | 0.12 |  |
| CIMP-high CRC | |  |  |  |  |  |  |
| No. of cases | | 46 | 48 | 49 | 57 |  |  |
| Multivariable-adjusted | | 1 (reference) | 1.08 (0.72, 1.62) | 1.13 (0.76, 1.70) | 1.41 (0.96, 2.09) | 0.13 |  |
| ***BRAF* mutation status** |  | |  |  |  |  | 0.47 |
| *BRAF*-wildtype CRC | |  |  |  |  |  |  |
| No. of cases | | 260 | 260 | 247 | 262 |  |  |
| Multivariable-adjusted | | 1 (reference) | 1.06 (0.89, 1.26) | 1.03 (0.86, 1.23) | 1.19 (0.99, 1.43) | 0.04 |  |
| *BRAF*-mutant CRC | |  |  |  |  |  |  |
| No. of cases | | 37 | 46 | 45 | 50 |  |  |
| Multivariable-adjusted | | 1 (reference) | 1.28 (0.83, 1.98) | 1.31 (0.85, 2.03) | 1.51 (0.99, 2.31) | 0.09 |  |
| ***KRAS* mutation status** |  | |  |  |  |  | 0.62 |
| *KRAS*-wildtype CRC | |  |  |  |  |  |  |
| No. of cases | | 170 | 171 | 147 | 172 |  |  |
| Multivariable-adjusted | | 1 (reference) | 1.06 (0.86, 1.31) | 0.94 (0.75, 1.18) | 1.19 (0.95, 1.48) | 0.14 |  |
| *KRAS*-mutant CRC | |  |  |  |  |  |  |
| No. of cases | | 114 | 124 | 126 | 120 |  |  |
| Multivariable-adjusted | | 1 (reference) | 1.16 (0.90, 1.50) | 1.20 (0.92, 1.56) | 1.27 (0.97, 1.66) | 0.06 |  |

^a^All analyses were stratified by age (in month), calendar year, and sex. Multivariable-adjusted hazard ratios were adjusted for body mass index (continuous with a ceiling at 35 kg/m^2^), physical activity (continuous with a ceiling at 50 metabolic equivalent task score-hours/week), smoking status (never, past, or current), regular use of aspirin or other non-steroidal anti-inflammatory drugs (≥2 tablets per week: yes or no), family history of colorectal cancer (yes or no), history of previous lower gastrointestinal endoscopy (yes or no), alcohol intake (continuous with a ceiling at 30 g/day), and total energy intake (continuous) .

^b^The healthy (or unhealthy) plant-based diet index was used as a continuous variable in the regression model except for individuals below 5th percentile and those above 95th percentile for whom the 5th and 95th percentile values, respectively, were used to eliminate outlier effects.

^c^We tested for heterogeneity by using a likelihood ratio test, comparing a multivariable-adjusted model that allows separate associations for different colorectal cancer subtypes with a model that assumes a common association.

Abbreviations: CIMP, CpG island methylator phenotype; CRC, colorectal cancer; MSI, microsatellite instability.

Supplementary Table 14. Hazard ratios with 95% confidence intervals of incident colon cancer and rectal cancer by tumor molecular features according to the healthy and unhealthy plant-based diet indices (per 15-unit, the difference between the median score values of extreme quartiles) in the pooled cohort

|  | Colon cancer | |  | Rectal cancer | |
| --- | --- | --- | --- | --- | --- |
|  | **Healthy plant-based diet index** | **Unhealthy plant-based diet index** |  | **Healthy plant-based diet index** | **Unhealthy plant-based diet index** |
| **MSI status** |  |  |  |  |  |
| Non-MSI-high |  |  |  |  |  |
| No. of cases | 745 | |  | 245 | |
| Multivariable-adjusted | 0.92 (0.75, 1.14) | 1.16 (0.94, 1.42) |  | 0.88 (0.61, 1.27) | 0.92 (0.63, 1.34) |
| MSI-high |  |  |  |  |  |
| No. of cases | 182 | |  | 7 | |
| Multivariable-adjusted | 0.85 (0.55, 1.32) | 1.56 (1.00, 2.43) |  | 2.18 (0.54, 8.84) | 1.06 (0.40, 2.85) |
| P heterogeneity | 0.75 | 0.22 |  | 0.22 | 0.78 |
| **CIMP status** |  |  |  |  |  |
| CIMP-low/negative |  |  |  |  |  |
| No. of cases | 708 | |  | 240 | |
| Multivariable-adjusted | 0.99 (0.80, 1.23) | 1.13 (0.91, 1.40) |  | 0.90 (0.62, 1.32) | 0.95 (0.65, 1.40) |
| CIMP-high |  |  |  |  |  |
| No. of cases | 192 | |  | 7 | |
| Multivariable-adjusted | 0.97 (0.65, 1.45) | 1.33 (0.88, 2.02) |  | 1,83 (0.36, 9.40) | 1.69 (0.41, 6.91) |
| P heterogeneity | 0.91 | 0.48 |  | 0.41 | 0.44 |
| ***BRAF* mutation status** |  |  |  |  |  |
| *BRAF*-wildtype |  |  |  |  |  |
| No. of cases | 766 | |  | 254 | |
| Multivariable-adjusted | 0.98 (0.80, 1.21) | 1.21 (0.98, 1.49) |  | 0.83 (0.59, 1.16) | 1.00 (0.69, 1.43) |
| *BRAF*-mutant |  |  |  |  |  |
| No. of cases | 167 | |  | 8 | |
| Multivariable-adjusted | 0.71 (0.47, 1.06) | 1.29 (0.85, 1.95) |  | 1.34 (0.27, 6.76) | 0.97 (0.42, 2.25) |
| P heterogeneity | 0.15 | 0.77 |  | 0.56 | 0.95 |
| ***KRAS* mutation status** |  |  |  |  |  |
| *KRAS*-wildtype |  |  |  |  |  |
| No. of cases | 503 | |  | 152 | |
| Multivariable-adjusted | 0.73 (0.57, 0.94) | 1.13 (0.89, 1.43) |  | 0.85 (0.55, 1.33) | 0.97 (0.59, 1.59) |
| *KRAS*-mutant |  |  |  |  |  |
| No. of cases | 380 | |  | 99 | |
| Multivariable-adjusted | 1.25 (0.93, 1.67) | 1.35 (0.99, 1.83) |  | 1.13 (0.66, 1.92) | 0.85 (0.51, 1.42) |
| P heterogeneity | 0.004 | 0.35 |  | 0.41 | 0.71 |

All analyses were stratified by age (in month), calendar year, and sex. Multivariable-adjusted hazard ratios were adjusted for body mass index (continuous with a ceiling at 35 kg/m^2^), physical activity (continuous with a ceiling at 50 metabolic equivalent task score-hours/week), smoking status (never, past, or current), regular use of aspirin or other non-steroidal anti-inflammatory drugs (≥2 tablets per week: yes or no), family history of colorectal cancer (yes or no), history of previous lower gastrointestinal endoscopy (yes or no), alcohol intake (continuous with a ceiling at 30 g/day), and total energy intake (continuous) . The inverse probability weighting method was applied to reduce selection bias due to molecular data availability.

Supplementary Table 15. Multivariable hazard ratios with 95% confidence intervals of incident colorectal cancer subclassified by *KRAS* mutation status according to individual plant food (per one serving/day) in the pooled cohort

|  | *KRAS*-wildtype  (n=660) | *KRAS*-mutant  (n=484) | P heterogeneity |
| --- | --- | --- | --- |
| **Healthy plant foods** |  |  |  |
| Whole grains | 0.92 (0.83, 1.02) | 1.07 (0.95, 1.22) | 0.05 |
| Fruits | 0.95 (0.85, 1.06) | 0.93 (0.82, 1.06) | 0.82 |
| Vegetables | 1.04 (0.96, 1.11) | 1.01 (0.93, 1.09) | 0.60 |
| Legumes | 1.09 (0.71, 1.66) | 0.74 (0.45, 1.21) | 0.22 |
| Nuts | 1.37 (0.92, 1.03) | 0.84 (0.53, 1.32) | 0.10 |
| Vegetable oils | 0.92 (0.67, 1.26) | 0.99 (0.68, 1.42) | 0.78 |
| Tea/coffee | 1.01 (0.95, 1.08) | 1.00 (0.93, 1.07) | 0.70 |
| **Unhealthy plant foods** |  |  |  |
| Refined grains | 1.10 (0.98, 1.23) | 0.95 (0.83, 1.08) | 0.08 |
| Sweets/desserts | 0.99 (0.88, 1.11) | 1.03 (0.91, 1.18) | 0.61 |
| Potatoes | 1.34 (0.95, 1.88) | 1.06 (0.71, 1.57) | 0.35 |
| Fruit juice | 1.02 (0.85, 1.21) | 0.87 (0.71, 1.07) | 0.26 |
| Sugar-sweetened beverages | 1.23 (0.91, 1.66) | 1.05 (0.74, 1.49) | 0.48 |

All analyses were stratified by age (in month), calendar year, and sex, and adjusted for body mass index (continuous with a ceiling at 35 kg/m^2^), physical activity (continuous with a ceiling at 50 metabolic equivalent task score-hours/week), smoking status (never, past, or current), regular use of aspirin or other non-steroidal anti-inflammatory drugs (≥2 tablets per week: yes or no), family history of colorectal cancer (yes or no), history of previous lower gastrointestinal endoscopy (yes or no), alcohol intake (continuous with a ceiling at 30 g/day), total energy intake (continuous), and intake of six animal food groups (continuous).

Supplementary Table 16. Hazard ratios with 95% confidence intervals of incident colorectal cancer according to the healthy and unhealthy plant-based diet indices (per 15-unit, the difference between the median score values of extreme quartiles) in the pooled cohort, stratified by age and body mass index

|  | Healthy plant-based diet index | Unhealthy plant-based diet index |
| --- | --- | --- |
| **Age** |  |  |
| <50 years | 1.11 (0.60, 2.05) | 1.07 (0.61, 1.88) |
| 50+ years | 0.90 (0.81, 0.99) | 1.16 (1.05, 1.28) |
| P-interaction | 0.46 | 0.87 |
| **Body mass index** |  |  |
| <25 kg/m^2^ | 0.95 (0.82, 1.11) | 1.11 (0.96, 1.29) |
| 25+ kg/m^2^ | 0.86 (0.75, 0.99) | 1.16 (1.01, 1.32) |
| P-interaction | 0.67 | 0.53 |

All analyses were stratified by age (in month), calendar year, and sex. Multivariable-adjusted hazard ratios were adjusted for body mass index (continuous with a ceiling at 35 kg/m^2^), physical activity (continuous with a ceiling at 50 metabolic equivalent task score-hours/week), smoking status (never, past, or current), regular use of aspirin or other non-steroidal anti-inflammatory drugs (≥2 tablets per week: yes or no), family history of colorectal cancer (yes or no), history of previous lower gastrointestinal endoscopy (yes or no), alcohol intake (continuous with a ceiling at 30 g/day), and total energy intake (continuous) . The healthy (or unhealthy) plant-based diet index was used as a continuous variable in the regression model except for individuals below 5th percentile and those above 95th percentile for whom the 5th and 95th percentile values, respectively, were used to eliminate outlier effects.

Supplementary Table 17. Hazard ratios with 95% confidence intervals of incident later-onset colorectal cancer (diagnosed before age 50) according to the healthy and unhealthy plant-based diet indices in the pooled cohort^a^

|  | Quartiles of healthy or unhealthy plant-based diet index | | | | P-trend^b^ |
| --- | --- | --- | --- | --- | --- |
|  | Quartile 1 | Quartile 2 | Quartile 3 | Quartile 4 |  |
| **Healthy plant-based diet index** | | | | | |
| No. of cases | 723 | 693 | 807 | 794 |  |
| Age-adjusted | 1 (reference) | 0.86 (0.77, 0.95) | 0.91 (0.82, 1.00) | 0.82 (0.74, 0.91) | 0.002 |
| Multivariable-adjusted | 1 (reference) | 0.86 (0.77, 0.96) | 0.92 (0.83, 1.02) | 0.85 (0.76, 0.95) | 0.04 |
| **Unhealthy plant-based diet index** | | | | | |
| No. of cases | 745 | 756 | 760 | 756 |  |
| Age-adjusted | 1 (reference) | 1.04 (0.94, 1.16) | 1.06 (0.96, 1.17) | 1.11 (1.00, 1.23) | 0.03 |
| Multivariable-adjusted | 1 (reference) | 1.07 (0.96, 1.19) | 1.09 (0.98, 1.21) | 1.16 (1.04, 1.30) | 0.004 |

^a^All analyses were stratified by age (in month), calendar year, and sex. Multivariable-adjusted hazard ratios were adjusted for body mass index (continuous with a ceiling at 35 kg/m^2^), physical activity (continuous with a ceiling at 50 metabolic equivalent task score-hours/week), smoking status (never, past, or current), regular use of aspirin or other non-steroidal anti-inflammatory drugs (≥2 tablets per week: yes or no), family history of colorectal cancer (yes or no), history of previous lower gastrointestinal endoscopy (yes or no), alcohol intake (continuous with a ceiling at 30 g/day), and total energy intake (continuous) . In NHS-only analyses, we also adjusted for postmenopausal hormone use (premenopausal, postmenopausal never, past, or current use).

^b^The healthy (or unhealthy) plant-based diet index was used as a continuous variable in the regression model except for individuals below 5th percentile and those above 95th percentile for whom the 5th and 95th percentile values, respectively, were used to eliminate outlier effects.

Supplementary Table 18. Hazard ratios with 95% confidence intervals of incident colorectal cancer according to the healthy or unhealthy plant-based diet index in the Nurses’ Health Study (NHS) and Health Professionals Follow-up Study (HPFS), stopped updating diet after the diagnosis of other disease outcomes^a^

|  | Quartiles of healthy or unhealthy plant-based diet index | | | | P-trend^b^ |
| --- | --- | --- | --- | --- | --- |
|  | Quartile 1 | Quartile 2 | Quartile 3 | Quartile 4 |  |
| **Healthy plant-based diet index** | | | | | |
| **NHS** |  |  |  |  |  |
| No. of cases | 415 | 369 | 445 | 459 |  |
| Multivariable-adjusted | 1 (reference) | 0.83 (0.72, 0.95) | 0.92 (0.80, 1.06) | 0.89 (0.77, 1.04) | 0.34 |
| **HPFS** |  |  |  |  |  |
| No. of cases | 329 | 340 | 362 | 358 |  |
| Multivariable-adjusted | 1 (reference) | 0.94 (0.81, 1.10) | 0.92 (0.79, 1.08) | 0.84 (0.71, 0.99) | 0.06 |
| **Pooled** |  |  |  |  |  |
| No. of cases | 744 | 709 | 807 | 817 |  |
| Multivariable-adjusted | 1 (reference) | 0.88 (0.79, 0.98) | 0.92 (0.83, 1.02) | 0.87 (0.78, 0.97) | 0.05 |
| **Unhealthy plant-based diet index** | | | | | |
| **NHS** |  |  |  |  |  |
| No. of cases | 401 | 430 | 417 | 440 |  |
| Multivariable-adjusted | 1 (reference) | 1.11 (0.96, 1.27) | 1.09 (0.94, 1.26) | 1.17 (1.01, 1.36) | 0.07 |
| **HPFS** |  |  |  |  |  |
| No. of cases | 355 | 354 | 342 | 338 |  |
| Multivariable-adjusted | 1 (reference) | 1.13 (0.97, 1.31) | 1.12 (0.95, 1.30) | 1.20 (1.02, 1.42) | 0.02 |
| **Pooled** |  |  |  |  |  |
| No. of cases | 756 | 784 | 759 | 778 |  |
| Multivariable-adjusted | 1 (reference) | 1.12 (1.01, 1.24) | 1.10 (0.99, 1.22) | 1.19 (1.07, 1.33) | 0.003 |

^a^All analyses were stratified by age (in month), calendar year, and sex. Multivariable-adjusted hazard ratios were adjusted for body mass index (continuous with a ceiling at 35 kg/m^2^), physical activity (continuous with a ceiling at 50 metabolic equivalent task score-hours/week), smoking status (never, past, or current), regular use of aspirin or other non-steroidal anti-inflammatory drugs (≥2 tablets per week: yes or no), family history of colorectal cancer (yes or no), history of previous lower gastrointestinal endoscopy (yes or no), alcohol intake (continuous with a ceiling at 30 g/day), and total energy intake (continuous) . In NHS-only analyses, we also adjusted for postmenopausal hormone use (premenopausal, postmenopausal never, past, or current use).

^b^The healthy (or unhealthy) plant-based diet index was used as a continuous variable in the regression model except for individuals below 5th percentile and those above 95th percentile for whom the 5th and 95th percentile values, respectively, were used to eliminate outlier effects.

Abbreviations: HPFS, Health Professionals Follow-up Study; NHS, Nurses’ Health Study.

Supplementary Table 19. Hazard ratios with 95% confidence intervals of incident colorectal cancer (CRC) by tumor molecular features according to the healthy or unhealthy plant-based diet indices in the pooled cohort, stopped updating diet after the diagnosis of other disease outcomes^a^

|  | | Quartiles of healthy or unhealthy plant-based diet index | | | | P-trend^b^ | P-heterogeneity^c^ |
| --- | --- | --- | --- | --- | --- | --- | --- |
|  | | Quartile 1 | Quartile 2 | Quartile 3 | Quartile 4 |  |  |
| **Healthy plant-based diet index** | | | | | | | |
| **MSI status** |  | |  |  |  |  | 0.97 |
| Non-MSI-high CRC | |  |  |  |  |  |  |
| No. of cases | | 234 | 257 | 264 | 246 |  |  |
| Multivariable-adjusted | | 1 (reference) | 1.06 (0.87, 1.29) | 0.96 (0.78, 1.17) | 0.85 (0.69, 1.06) | 0.06 |  |
| MSI-high CRC | |  |  |  |  |  |  |
| No. of cases | | 49 | 37 | 54 | 50 |  |  |
| Multivariable-adjusted | | 1 (reference) | 0.57 (0.36, 0.89) | 0.87 (0.57, 1.31) | 0.73 (0.48, 1.12) | 0.40 |  |
| **CIMP status** |  | |  |  |  |  | 0.71 |
| CIMP-low/negative CRC | |  |  |  |  |  |  |
| No. of cases | | 217 | 241 | 259 | 243 |  |  |
| Multivariable-adjusted | | 1 (reference) | 1.05 (0.86, 1.29) | 1.02 (0.83, 1.25) | 0.92 (0.74, 1.14) | 0.27 |  |
| CIMP-high CRC | |  |  |  |  |  |  |
| No. of cases | | 50 | 41 | 54 | 55 |  |  |
| Multivariable-adjusted | | 1 (reference) | 0.70 (0.46, 1.08) | 0.95 (0.62, 1.45) | 0.86 (0.56, 1.30) | 0.90 |  |
| ***BRAF* mutation status** |  | |  |  |  |  | 0.34 |
| *BRAF*-wildtype CRC | |  |  |  |  |  |  |
| No. of cases | | 238 | 252 | 278 | 261 |  |  |
| Multivariable-adjusted | | 1 (reference) | 1.01 (0.83, 1.23) | 1.00 (0.82, 1.22) | 0.87 (0.71, 1.07) | 0.11 |  |
| *BRAF*-mutant CRC | |  |  |  |  |  |  |
| No. of cases | | 46 | 45 | 46 | 41 |  |  |
| Multivariable-adjusted | | 1 (reference) | 0.87 (0.56, 1.35) | 0.78 (0.49, 1.24) | 0.68 (0.43, 1.09) | 0.08 |  |
| ***KRAS* mutation status** |  | |  |  |  |  | 0.006 |
| *KRAS*-wildtype CRC | |  |  |  |  |  |  |
| No. of cases | | 162 | 185 | 164 | 149 |  |  |
| Multivariable-adjusted | | 1 (reference) | 1.06 (0.84, 1.34) | 0.84 (0.66, 1.08) | 0.73 (0.57, 0.94) | 0.003 |  |
| *KRAS*-mutant CRC | |  |  |  |  |  |  |
| No. of cases | | 109 | 95 | 140 | 140 |  |  |
| Multivariable-adjusted | | 1 (reference) | 0.88 (0.65, 1.19) | 1.12 (0.84, 1.49) | 1.06 (0.79, 1.42) | 0.35 |  |
| **Unhealthy plant-based diet index** | | | | | | | |
| **MSI status** |  | |  |  |  |  | 0.33 |
| Non-MSI-high CRC | |  |  |  |  |  |  |
| No. of cases | | 246 | 261 | 258 | 236 |  |  |
| Multivariable-adjusted | | 1 (reference) | 1.07 (0.88, 1.30) | 1.16 (0.96, 1.41) | 1.09 (0.89, 1.34) | 0.24 |  |
| MSI-high CRC | |  |  |  |  |  |  |
| No. of cases | | 46 | 42 | 39 | 63 |  |  |
| Multivariable-adjusted | | 1 (reference) | 1.01 (0.64, 1.60) | 0.87 (0.55, 1.36) | 1.50 (0.99, 2.26) | 0.13 |  |
| **CIMP status** |  | |  |  |  |  | 0.54 |
| CIMP-low/negative CRC | |  |  |  |  |  |  |
| No. of cases | | 234 | 256 | 243 | 227 |  |  |
| Multivariable-adjusted | | 1 (reference) | 1.10 (0.90, 1.34) | 1.09 (0.89, 1.33) | 1.09 (0.88, 1.35) | 0.38 |  |
| CIMP-high CRC | |  |  |  |  |  |  |
| No. of cases | | 51 | 46 | 44 | 59 |  |  |
| Multivariable-adjusted | | 1 (reference) | 0.99 (0.66, 1.49) | 1.05 (0.69, 1.61) | 1.34 (0.89, 2.00) | 0.28 |  |
| ***BRAF* mutation status** |  | |  |  |  |  | 0.70 |
| *BRAF*-wildtype CRC | |  |  |  |  |  |  |
| No. of cases | | 261 | 260 | 255 | 253 |  |  |
| Multivariable-adjusted | | 1 (reference) | 1.06 (0.87, 1.28) | 1.09 (0.90, 1.32) | 1.15 (0.95, 1.41) | 0.11 |  |
| *BRAF*-mutant CRC | |  |  |  |  |  |  |
| No. of cases | | 38 | 43 | 45 | 52 |  |  |
| Multivariable-adjusted | | 1 (reference) | 1.05 (0.65, 1.71) | 1.26 (0.78, 2.04) | 1.34 (0.85, 2.12) | 0.25 |  |
| ***KRAS* mutation status** |  | |  |  |  |  | 0.40 |
| *KRAS*-wildtype CRC | |  |  |  |  |  |  |
| No. of cases | | 172 | 167 | 151 | 170 |  |  |
| Multivariable-adjusted | | 1 (reference) | 1.04 (0.82, 1.33) | 0.98 (0.77, 1.24) | 1.13 (0.89, 1.42) | 0.55 |  |
| *KRAS*-mutant CRC | |  |  |  |  |  |  |
| No. of cases | | 115 | 122 | 130 | 117 |  |  |
| Multivariable-adjusted | | 1 (reference) | 1.02 (0.77, 1.35) | 1.23 (0.93, 1.62) | 1.21 (0.91, 1.63) | 0.11 |  |

^a^All analyses were stratified by age (in month), calendar year, and sex. Multivariable-adjusted hazard ratios were adjusted for body mass index (continuous with a ceiling at 35 kg/m^2^), physical activity (continuous with a ceiling at 50 metabolic equivalent task score-hours/week), smoking status (never, past, or current), regular use of aspirin or other non-steroidal anti-inflammatory drugs (≥2 tablets per week: yes or no), family history of colorectal cancer (yes or no), history of previous lower gastrointestinal endoscopy (yes or no), alcohol intake (continuous with a ceiling at 30 g/day), and total energy intake (continuous) . The inverse probability weighting method was applied to reduce selection bias due to molecular data availability.

^b^The healthy (or unhealthy) plant-based diet index was used as a continuous variable in the regression model except for individuals below 5th percentile and those above 95th percentile for whom the 5th and 95th percentile values, respectively, were used to eliminate outlier effects.

^c^We tested for heterogeneity by using a likelihood ratio test, comparing a multivariable-adjusted model that allows separate associations for different colorectal cancer subtypes with a model that assumes a common association.

Abbreviations: CIMP, CpG island methylator phenotype; CRC, colorectal cancer; MSI, microsatellite instability.

Supplementary Table 20. Pearson correlations between plant-based diet indices and other dietary indices in the pooled cohorts

|  | hPDI | uPDI | PDI | Prudent Diet | DASH | AMED | EDIP | EDIH |
| --- | --- | --- | --- | --- | --- | --- | --- | --- |
| hPDI | 1 | -0.35 | 0.26 | 0.35 | 0.59 | 0.42 | -0.33 | -0.55 |
| uPDI |  | 1 | -0.09 | -0.65 | -0.57 | -0.60 | 0.20 | -0.00 |
| PDI |  |  | 1 | 0.50 | 0.41 | 0.54 | -0.10 | -0.07 |
| Prudent Diet |  |  |  | 1 | 0.72 | 0.73 | -0.07 | -0.02 |
| DASH |  |  |  |  | 1 | 0.73 | -0.24 | -0.32 |
| AMED |  |  |  |  |  | 1 | -0.16 | -0.14 |
| EDIP |  |  |  |  |  |  | 1 | 0.65 |
| EDIH |  |  |  |  |  |  |  | 1 |

Abbreviations: AMED, Alternative Mediterranean Diet; DASH, Dietary Approaches to Stop Hypertension; EDIH, empirical dietary index for hyperinsulinemia; EDIP, empirical dietary inflammatory pattern; hPDI, healthy plant-based diet index; PDI, plant-based diet index; uPDI, unhealthy plant-based diet index.


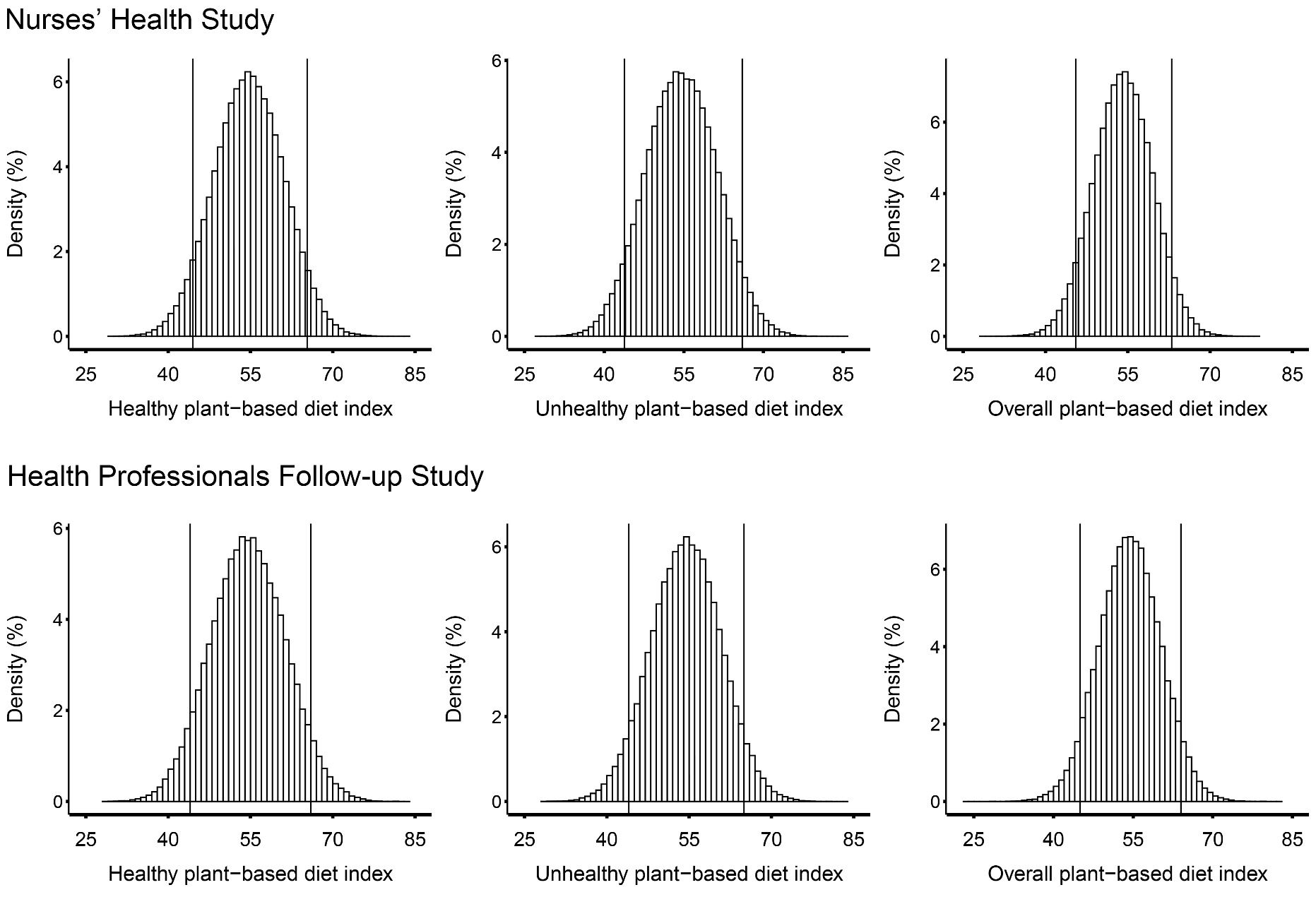


Supplementary Figure 1. Distribution of the three plant-based diet indices in the Nurses’ Health Study and the Health Professionals Follow-up Study. The left and right vertical lines indicate the 5th and 95th percentile values, respectively.


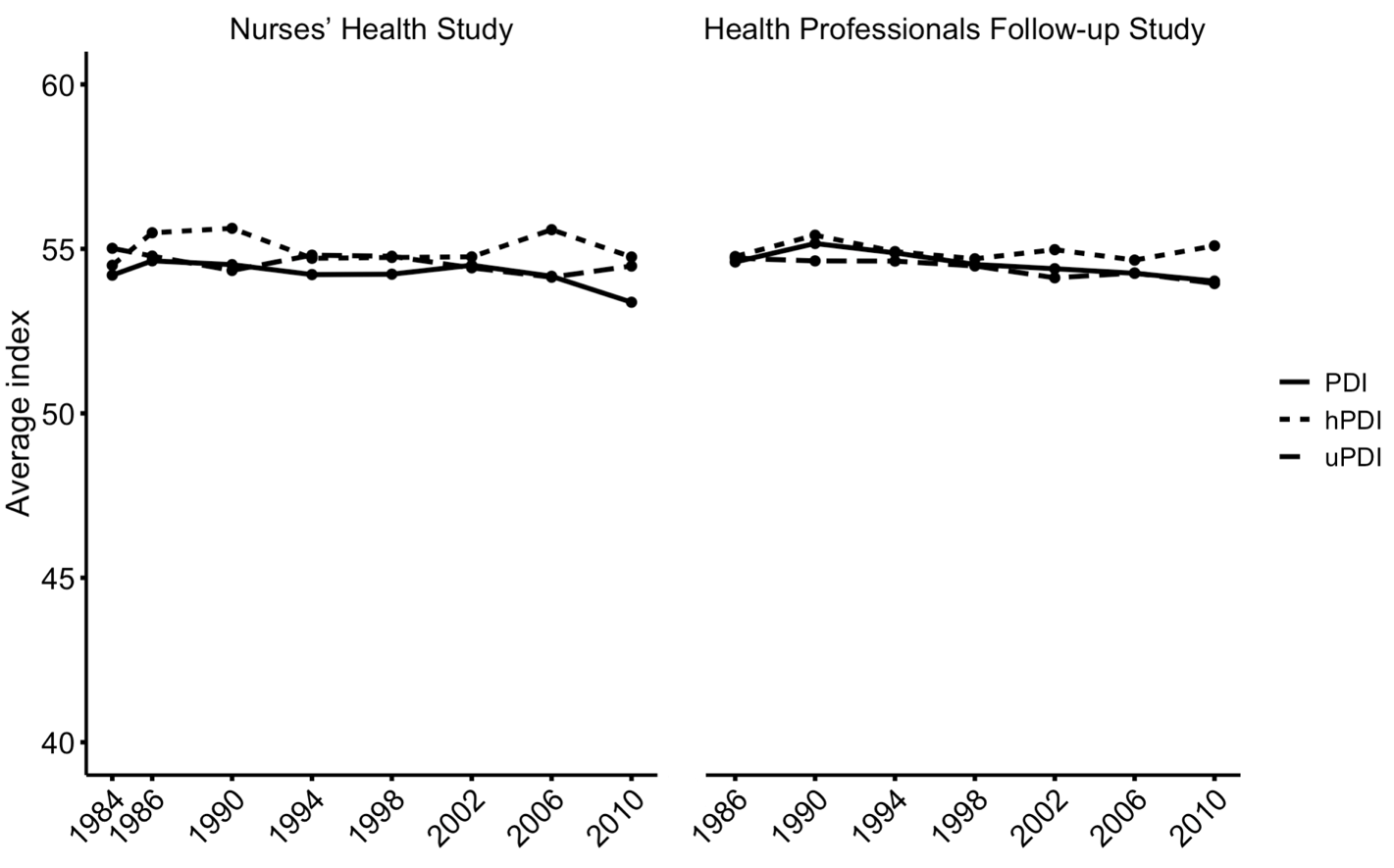


Supplementary Figure 2. Average values of the three plant-based diet indices in the two cohorts across the follow-up period. Abbreviations: hPDI, healthy plant-based diet index; PDI, plant-based diet index; uPDI, unhealthy plant-based diet index.
